# The Distance between Minima of Electron Density and Electrostatic Potential as a Measure of Halogen Bond Strength

**DOI:** 10.3390/molecules27154848

**Published:** 2022-07-28

**Authors:** Edem R. Chakalov, Elena Yu. Tupikina, Daniil M. Ivanov, Ekaterina V. Bartashevich, Peter M. Tolstoy

**Affiliations:** 1 Institute of Chemistry, St. Petersburg State University, 198504 St. Petersburg, Russia; st086266@student.spbu.ru (E.R.C.); e.tupikina@spbu.ru (E.Y.T.); 2 Chemistry Department, South Ural State University, 454080 Chelyabinsk, Russia; bartashevichev@susu.ru

**Keywords:** halogen bond, QTAIM, electron density, electrostatic potential, interaction energy, bond strength, density functional theory, phosphine oxide, ^31^P NMR

## Abstract

In this study, we present results of a detailed topological analysis of electron density (ED) of 145 halogen-bonded complexes formed by various fluorine-, chlorine-, bromine-, and iodine-containing compounds with trimethylphosphine oxide, Me_3_PO. To characterize the halogen bond (XB) strength, we used the complexation enthalpy, the interatomic distance between oxygen and halogen, as well as the typical set of electron density properties at the bond critical points calculated at B3LYP/jorge-ATZP level of theory. We show for the first time that it is possible to predict the XB strength based on the distance between the minima of ED and molecular electrostatic potential (ESP) along the XB path. The gap between ED and ESP minima exponentially depends on local electronic kinetic energy density at the bond critical point and tends to be a common limiting value for the strongest halogen bond.

## 1. Introduction

**Figure 1:**
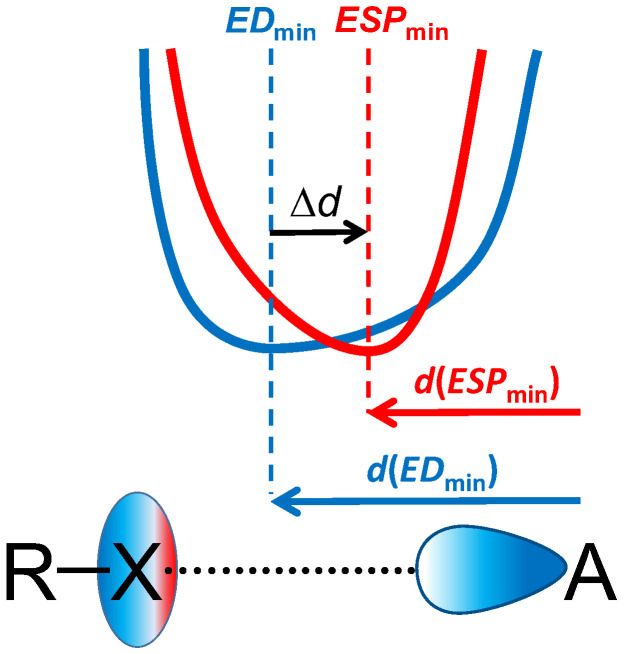
Schematic representation of a halogen bond (X—halogen atom, A—nucleophilic site), ED (blue) and ESP (red) distributions along the bond path. The graphical definition of *d*(*ED*_min_) (blue), *d*(*ESP*_min_) (red), and Δ*d* (black) are given.

Among various types of *σ*-hole interactions, halogen bonds (XBs) are among the most known and widely investigated [1,2,3,4]. XBs were shown to play a significant role in organocatalysis [5,6,7,8], crystal engineering and supramolecular chemistry [9,10,11,12,13], materials science [14,15,16,17], stabilization of explosives [18], drug design [19,20], etc. XBs R–X⋯A (X—halogen) are formed between an electron-depleted region on the continuation of the R–X bond and an electron-rich region of another atom or molecule A (Figure 1, bottom). Main geometric criteria of a XB formation are the short X⋯A distance, smaller than the sum of X and A van der Waals radii, and the proximity of the halogen bond angle to the linear [21]. As an electronic criterion, the presence of a critical point of type (3; −1) (bond critical point, BCP) for calculated [22,23,24] or experimentally measured electron density (ED) [25,26,27,28] along the X⋯A bond path in QTAIM analysis (Quantum Theory of Atoms in Molecules [29]) is often used.

The philicity of halogen bonding participants can be determined using geometric criterion (the angle around electrophilic site is close to 180°) [21], spectral manifestations (for instance, in ultraviolet–visible (UV–vis) spectra [30,31,32,33] or using calculated parameters (electron density deformation (EDD) [34,35,36,37,38,39,40], electron localization function (ELF) distribution [41,42,43,44], natural bond orbital charge transfer [45,46], sums of atomic charges [47], and molecular electrostatic potential (ESP) distribution on the van der Waals surface with *ρ*(*r*) = 0.001 e/Bohr^3^, in representative planes or along the bond path [48,49,50,51,52,53,54,55]).

In [54], it was mentioned for the first time for a series of hydrogen-bonded complexes that the relative arrangement of ED and ESP minima positions along the hydrogen bond path is the same for all complexes. Moreover, the superposition of gradient fields of ED and ESP as discussed for different intermolecular interactions is solids, including the studies based on experimental charge density [56,57]. These observations formed the basis of electronic criterion proposed in Ref. [24], which makes it possible to unambiguously determine the type of electrostatically driven noncovalent bonds. In accordance with electronic criterion, the minimum of ESP along the bond path is located closer to the atom that donates electrons, whereas the minimum of ED is located closer to the atom that delivers its electrophilic site for noncovalent bonding (Figure 1). The latter atom prescribes the name of “-ogen bonds” (e.g., hydrogen, halogen, chalcogen, pnictogen, etc.).

While for R–X⋯A XBs it is often clear which atom acts as an electrophile and which one acts as a nucleophile, the information value of the relative positions of ED and ESP minima seems to hold for less trivial cases of halogen-halogen contacts [58,59,60] and also beyond the halogen bonding in general [61,62].

Up to now, it is not known if there is an information value in the distance between ED and ESP minima, Δ*d*, defined as

(1) $$\Delta d=d\left(ED_{\min}\right)-d\left(ESP_{\min}\right)$$

(see also the graphical definition in Figure 1). A question arises: does a robust correlation exist between Δ*d* and such XB properties as its strength or the X⋯A distance? To the best of our knowledge, it is an original question, not yet discussed in the QTAIM-related literature. t Of fundamental interest, we expect that this sort of information could be useful for studying XBs in solids, where ED and ESP distributions could in principle be measured experimentally [63], whereas the direct experimental evaluation of XB energy is very difficult or even not possible.

**Figure 2:**
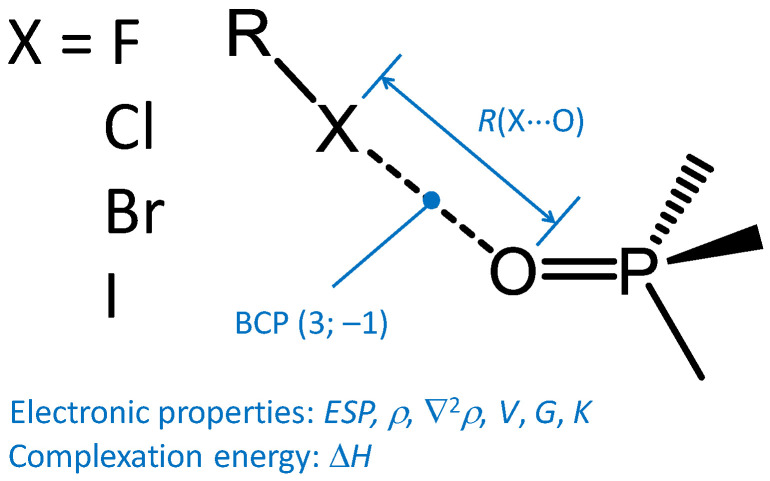
Schematic representation of halogen-bonded complexes formed between R–X (X = F, Cl, Br and I) and Me_3_PO. In blue are given geometric (interatomic distance *R*(X⋯O)), energetic (complexation enthalpy Δ*H*), and electronic (*ρ* stands for electron density, ∇^2^*ρ* for its Laplacian, *V*, *G* and *K* stand for local electron potential, kinetic and total energy densities at the bond critical point, BCP) parameters that were correlated with Δ*d* in this work (see Figure 1 for the definition of Δ*d*).

In this study, we checked the information value of Δ*d* for a series of 145 halogen-bonded complexes formed by various halogen donors with trimethylphosphine oxide, Me_3_PO (Figure 2) at the B3LYP/jorge-ATZP level of theory. The Me_3_PO molecule was chosen as a “standard” halogen acceptor, in a sense following the approach started many years ago by Gutmann and Beckett [64,65] and explored in recent years by a number of authors [66,67,68]. In order to reduce the number of internal degrees of freedom in the electron donor, we have selected Me_3_PO instead of Et_3_PO, which was originally used in the Gutmann–Beckett method. As halogen donors the molecules belonging to the following classes of F-, Cl-, Br- and I-containing compounds were considered: halogens, interhalides, oxohalides, pseudohalides, halogenated methane, ethylene, acetylene, benzene, phosgene and their derivatives, as well as thionyl- and sulfurylhalides, sulfur halides and sulfur hypohalites, and several halogenated nitrogen-containing inorganic compounds and some others. This choice of model systems makes it possible to track how a change in the halogen bond acceptor (i.e., its electronic properties as well as belonging to a certain class of chemical compounds) changes the properties of the halogen bond when the halogen bond acceptor Me_3_PO is fixed. The full list of halogen donors is given in Figure S1 in Supplementary Materials.

Previously, we have considered a similar but somewhat smaller set of complexes in order to build a correlation between the XB strength/geometry and the changes of two spectral parameters upon complexation: the ^31^P NMR chemical shift and the ν(P=O) stretching frequency [69] (similar correlations for hydrogen-bonded complexes with Me_3_PO were recently published as well [70]). We have shown that decent correlations of this kind do exist, and they could be fitted by simple analytical functions. Now, we turn our attention to electronic properties, such as Δ*d*, which is experimentally accessible—if the conditions are right—for single crystal samples. Several XB parameters were considered in this work and correlated with Δ*d*: the complexation enthalpy Δ*H*, the X⋯O interatomic distance *R*(X⋯O), and various parameters at XB electron density critical point of type (3; −1): (molecular electrostatic potential *ESP*(*r*_BCP_), electron density *ρ*(*r*_BCP_), Laplacian of electron density ∇^2^*ρ*(*r*_BCP_), local electron kinetic *G*(*r*_BCP_) and potential *V*(*r*_BCP_) energy densities and total electron energy density *K*(*r*_BCP_)). We also checked if ^31^P NMR chemical shifts of Me_3_PO correlate with Δ*d*.

## 2. Results and Discussion

**Figure 3:**
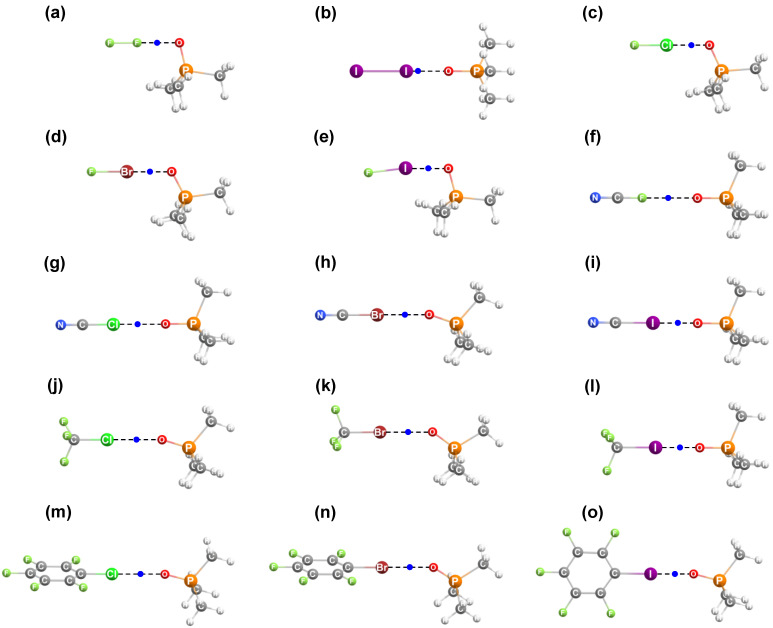
Some examples of optimized geometries of halogen-bonded complexes formed between (**a**) F_2_; (**b**) I_2_; (**c**) ClF; (**d**) BrF; (**e**) IF; (**f**) FCN; (**g**) ClCN; (**h**) BrCN; (**i**) ICN; (**j**) CF_3_Cl; (**k**) CF_3_Br; (**l**) CF_3_I; (**m**) C_6_F_5_Cl; (**n**) C_6_F_5_Br; (**o**) C_6_F_5_I and Me_3_PO considered in this work. Blue dot marks the position of the X⋯O bond critical point.

The optimized geometries of some representative examples of halogen-bonded complexes belonging to different classes of inorganic and organic compounds are shown in Figure 3 and all 145 halogen-bonded complexes are shown in Supplementary Materials (Figure S1). The majority of XBs are linear (or close to linear) and formed along the direction of expected oxygen lone pair localization. The numerical values of relevant parameters are listed in Supplementary Materials: geometric, energetic and spectroscopic parameters (Table S1), and QTAIM electronic parameters (Table S2).

**Figure 4:**
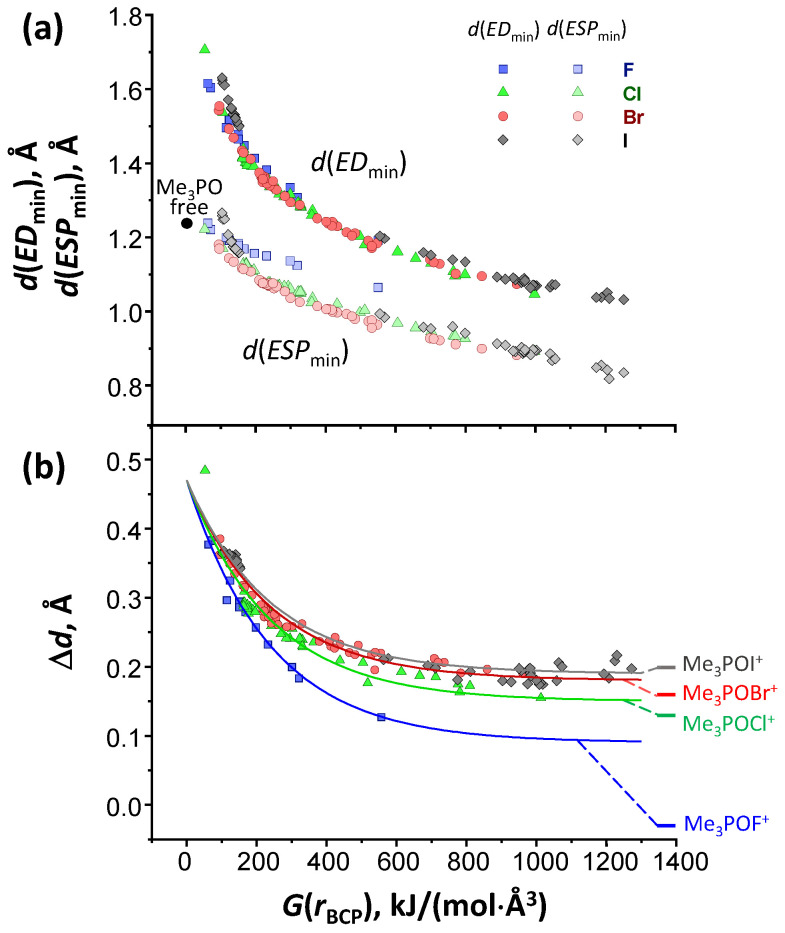
(**a**) Distances from oxygen atom to minima of molecular electrostatic potential *d*(*ESP*_min_) (lighter colors) and electron density *d*(*ED*_min_) (darker colors) along the X⋯O (X = F, Cl, Br and I) bond path; (**b**) distances between ED and ESP minima Δ*d* as a function of *G*(*r*_BCP_) for a series of halogen-bonded complexes formed between R–X and Me_3_PO. The solid curves correspond to Equation (2) with fitted parameters listed in Table 1.

Figure 4a shows the dependence of distances from oxygen to ESP and ED minima—*d*(*ESP*_min_) and *d*(*ED*_min_), respectively—along the XB path as a function of the local electron kinetic energy density at critical point of type (3; −1), *G*(*r*_BCP_) (in kJ/(mol⋅Å^3^)), taken as a measure of XB strength. In Figure S2 (Supplementary Materials) we show several examples of ED and ESP profiles along the bond path for weak, medium, and strong R–Cl⋯OPMe_3_ complexes. The *d*(*ESP*_min_) distance is among the largest ones for free Me_3_PO (see the black dot in Figure 4a) and falls upon the increase in the XB strength. The *d*(*ED*_min_) is obviously infinite for free Me_3_PO, but rapidly—quasi-exponentially—decreases when the XB gets stronger. The *d*(*ESP*_min_) and *d*(*ED*_min_) values appear to be almost halogen-independent, except for complexes with F-donors, the *d*(*ESP*_min_) data points for which deviate consistently from the other sets.

However, a closer look at the Δ*d* = *d*(*ESP*_min_) − *d*(*ED*_min_) values (Figure 4b) reveals that there are some systematic differences between complexes with Cl-, Br-, and I-donors as well. The general trend is the same for all halogens: the stronger is the XB, the closer are the ESP and ED minima. The Δ*d* values within each series seem to approach a limiting (asymptotic) value for the strongest XBs. A question arises, what are the limiting Δ*d* values for hypothetical strongest possible bonds? As models for such X⋯O bonds, we took cations Me_3_POX^+^, the optimized geometries of which together with the X⋯O bond parameters are shown in Figure S3. For these cations, the “asymptotic” *G*(*r*_BCP_) values are the largest and Δ*d* values are the smallest within the respective series of complexes (except for complexes with iodine, where there is a significant scattering of data points for strong XBs). We have added Δ*d* asymptotes as horizontal bold lines in Figure 4b. For F-donors, the Δ*d* even gets slightly negative, suggesting that the electrophile/nucleophile roles are practically swapped for the F–O bond in Me_3_POF^+^, as compared to non-covalent R–X⋯OPMe_3_ complexes (i.e., the F–O bond in Me_3_POF^+^ cannot be classified as a halogen bond).

The data sets in the Δ*d* plot versus *G*(*r*_BCP_) for the studied sets of halogen-bonded complexes could be reasonably well fitted, assuming an overall exponential behavior:

(2) $$\Delta d=a_{X}+\left(\Delta d_{0}-a_{X}\right)\cdot \exp\left(-G\left(r_{\mathrm{BCP}}\right)/b\right).$$

Interestingly, only one fitting parameter in Equation (2), $a_{X}$, is halogen-dependent (see Table 1). The rate of exponential fall *b* = 240 kJ/(mol⋅Å^3^) and the limiting Δ*d* value for *G*(*r*_BCP_) = 0 kJ/(mol⋅Å^3^), Δ*d*_0_ = 0.47 Å, seem to be virtually the same for all halogens. At the moment, it is not clear if these values for Δ*d*_0_ and *b* reflect some property of Me_3_PO or a property of XBs in general. The result of the fitting is added to Figure 4b as solid lines. Another noteworthy thing is that the fitting with Equation (2) does not reproduce the asymptotic values for Me_3_POX^+^ at *G*(*r*_BCP_) → ∞, though the relative position of the fitting asymptotes for F, Cl, Br and I is the same as for the corresponding cations Me_3_POX^+^. Solving Equation (2) for *G*(*r*_BCP_) one obtains

(3) $$G\left(r_{\mathrm{BCP}}\right)=b\cdot \ln\left(\frac{\Delta d_{0}-a_{X}}{\Delta d-a_{X}}\right).$$

It should be noted that Equation (3) makes sense only when the logarithm is defined (Δ*d > a*_X_) and positive (Δ*d* < Δ*d*_0_). This means that Equation (3) might become inapplicable for extremely weak and extremely strong XBs. Subsequently, the *G*(*r*_BCP_) values could be converted into the XB “complexation energies” Δ*E* (the difference of total electronic energies of the complex and its isolated relaxed constituents) using previously published halogen-dependent proportionality coefficients *k* (see the last column in Table 1; the values were taken from Table 2 in Ref. [69]),

(4) $$\Delta E=k\cdot G\left(r_{\mathrm{BCP}}\right).$$

**Figure 5:**
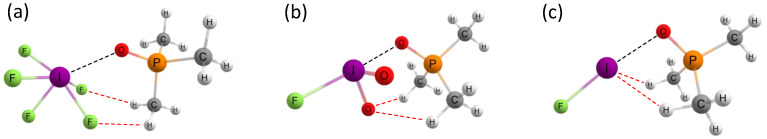
Examples of additional non-covalent interactions (red dashed lines) present in some halogen-bonded (black dashed line) R–X⋯OPMe_3_ complexes: hydrogen bonds between methyl protons of the Me_3_PO moiety and electronegative atoms of the halogen donor; (**а**) two H-bonds with two proton acceptors; (**b**) two H-bonds with one proton acceptor; (**c**) two H-bonds with the halogen donating atom.

At this point, it is worth making a brief comment concerning various quantitative models based on the properties of electron density at the bond critical points, which were previously successfully used for characterization of the energy for various types of noncovalent bonds [70,71,72,73,74,75]. Figure S4 shows the correlation between the complexation enthalpy Δ*H* and *G*(*r*_BCP_). Note that there is a significant scattering of the data points due to the fact that a number of complexes within the studied series are held not only by an XB, but also by other noncovalent interactions, most prominently weak hydrogen bonds between electronegative atoms of the halogen donor (including the halogen itself) and the methyl protons of the Me_3_PO moiety (Figure 5). For completeness of the subject, in Figures S5–S8 we show also correlations of electron density *ρ*(*r*_BCP_), Laplacian of electron density ∇^2^*ρ*(*r*_BCP_) and total electron energy density *K*(*r*_BCP_) = *G*(*r*_BCP_) + *V*(*r*_BCP_) with *d*(*ESP*_min_), *d*(*ED*_min_), Δ*d*, and *R*_norm_, as well as *G*(*r*_BCP_) correlation with *V*(*r*_BCP_), *ρ*(*r*_BCP_), ∇^2^*ρ*(*r*_BCP_) and *ESP*(*r*_BCP_). In turn, Figure S9 shows Δ*H*, *ρ*(*r*_BCP_), ∇^2^*ρ*(*r*_BCP_) and *ESP*(*r*_BCP_) dependence on Δ*d*.

**Figure 6:**
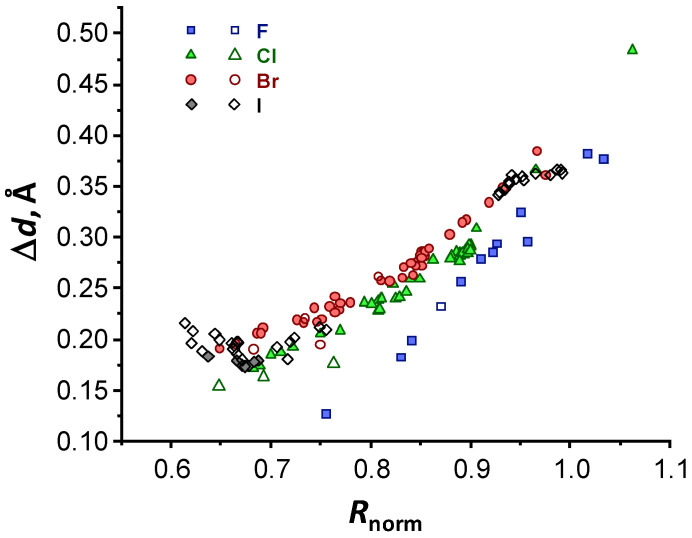
Distances between ED and ESP minima Δ*d* as a function of normalized interatomic distances *R*_norm_ between X (X = F, Cl, Br and I) and O (see definition in Equation (5)).

Finally, the Δ*d* values are plotted in Figure 6 as a function of *R*_norm_, defined as X⋯O interatomic distance, normalized by the sum of van der Waals radii (*R*_vdW_) of X (X = F, Cl, Br and I) and O:

(5) $$R_{\mathrm{norm}}=\frac{R\left(X\cdots O\right)}{R_{\mathrm{vdW}}\left(X\right)+R_{\mathrm{vdW}}\left(O\right)},$$

where the following values of the van der Waals radii were used: 1.52 Å (O), 1.47 Å (F), 1.75 Å (Cl), 1.85 Å (Br), 1.98 Å (I) [76]. The usage of normalized and unitless *R*_norm,_ instead of direct interatomic distances X⋯O, allows one—at least in principle—to compare XBs with participation of different halogens (see the dependence of absolute *R*(X⋯O) distances and *R*_norm_ on *G*(*r*_BCP_) in Figure S10). The data sets plotted in Figure 6 indicate slightly non-linear dependencies of Δ*d* on *R*_norm_ for each halogen, but generally show the same trends as Figure 4b, because the terms halogen bond strength and shortness are almost interchangeable: stronger/shorter XBs are characterized by smaller Δ*d* values.

To summarize the results so far, there is indeed a correlation between Δ*d* and XB energy and length. The exponential fall of Δ*d* upon strengthening/shortening of the XB is dependent on the type of halogen and most probably on the type of the electron-donating atom as well (oxygen in our case), but for a fixed pair of atoms the correlations seem to be largely independent on the substituents, which makes Δ*d* a promising tool in characterization of XBs and—speculatively—other types of non-covalent interactions as well.

Now we turn attention to the spectroscopic manifestation of halogen bonding within the studied series of complexes. In Ref. [69], some of us have demonstrated that isotropic ^31^P NMR chemical shift of Me_3_PO could be used as a spectroscopic marker for the halogen-donating ability of a probed molecule or, in other words, as a measure of the XB strength (length) in a R–X⋯OPMe_3_ complex. For the data sets presented in this work, reasonable correlations between the change of the ^31^P NMR chemical shift upon complexation Δ*δ*^31^P and the complexation enthalpy Δ*H* exist for chlorine- and bromine-containing complexes (Figure S11a). For fluorine- and iodine-containing ones, there is a large scattering of the data points, which in case of complexes with iodine is likely to be—at least partially—due to the presence of additional non-covalent interactions and, consequently, the influence of several competing factors (e.g., presence of additional interactions) on the electron shells of the phosphorus atom. Indeed, the Δ*δ*^31^P correlation with *G*(*r*_BCP_) values (Figure S11b) is noticeably better, though with significant residual scattering still (it should be mentioned, that the shape of Δ*δ*^31^P(Δ*d*) remains the same upon the change of a basis set).

**Figure 7:**
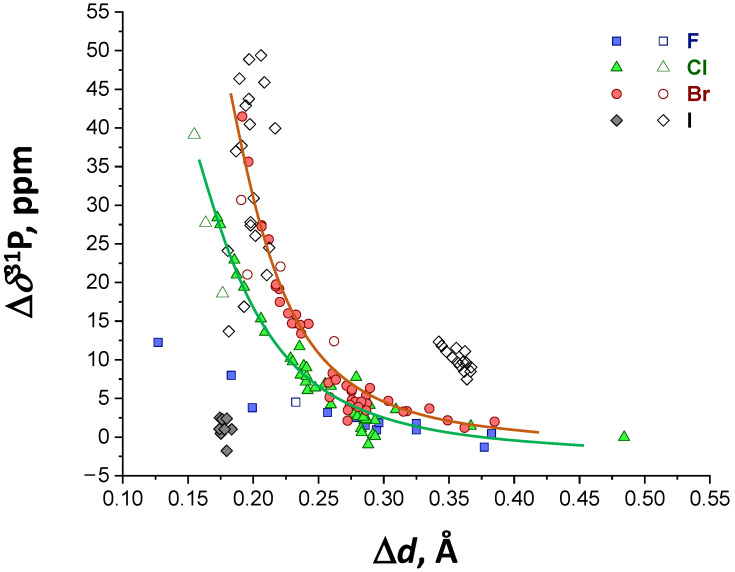
The correlation of Δ*δ*^31^P on Δ*d* for a series of R–X⋯OPMe_3_ complexes. The solid lines are guides for the eye added for Cl- and Br-bonded complexes.

Figure 7 shows the correlation of Δ*δ*^31^P with Δ*d*, which has a similar degree of data point scattering as Figure S11b. Because of that, we have added to Figure 7 two trend curves for chlorine- and bromine-containing complexes only. These curves serve only as guides for the eye, as it seems premature to propose a functional fit. Still, one could confirm that ^31^P NMR chemical shift sensitively reflect the changes in the electronic structure of the complexes—including the parameter in focus of this work, Δ*d*—and could serve for the characterization of XBs with phosphine oxides.

## 3. Computational Methods

The full geometry optimization, harmonic vibrational frequencies and NMR calculations were performed for studied complexes in vacuum using B3LYP functional [77] and nonrelativistic all-electron augmented triple-zeta valence quality basis set with polarization functions jorge-ATZP [78,79,80] (adopted from the Basis Set Exchange site [81]) using Gaussian16 software package [82]. Geometry optimization was performed using the default for Gaussian16 Berny algorithm without any geometry restrictions and standard convergence criteria (maximum/RMS forces and displacements of atoms smaller than 0.000450 a.u./0.000300 a.u. and 0.001800 a.u./0.001200 a.u., respectively). The optimized geometries were checked for the absence of imaginary vibrational frequencies. The presence of halogen bonds in the calculated complexes was confirmed by the criteria for halogen bond formation, according to the IUPAC recommendation [21]. For each halogen donor, only one optimized structure of its complex with phosphine oxide was considered. For a subset of complexes, we have checked that variations in the initial geometries lead to the same optimized structures. Because of this, we believe that they are true global minima. However, even if it is not the case, any local true minimum containing a halogen bond and satisfying the Virial theorem would be legitimate structure to perform topological analysis of electron density and use the resulting data to construct correlations.

The complexation enthalpy Δ*H* was calculated at 298.15 K as the enthalpy required to separate the interacting molecules at infinite distance (including the relaxation of monomers). Note that in this way the Δ*H* values include contributions not only from the XB, but also from any other non-covalent interactions which might be present between monomers of Me_3_PO and XB donor.

Isotropic NMR shielding constants *σ* were calculated using the gauge-independent atomic orbital (GIAO) approach [83] and converted into changes of chemical shifts upon complexation as Δ*δ*^31^P = *σ*_free_ − *σ*, where *σ*_free_ is the ^31^P nuclear shielding constant for an isolated Me_3_PO molecule.

The topological analysis of ED and ESP along the XB path was carried out within the framework of QTAIM methodology from the wave function files using MultiWFN software (http://sobereva.com/multiwfn/ accessed on 22 June 2022; Beijing Kein Research Center for Natural Sciences; Beijing, China; version 3.3.8) [84]. The ED and ESP minima positions from the oxygen atom along the XB path were determined with the following path searching parameters: maximum number of points of a path is 100000, with stepsize of 0.0005 Bohr and generation stop threshold of 0.005 Bohr distance to any critical point. This takes into account that for all studied complexes *d*(*ESP*_min_) < *d*(*ED*_min_), the definition given in Equation (1) makes all Δ*d* values positive.

Visualization of the studied complexes was performed using the Chemcraft software (available online at www.chemcraftprog.com accessed on 22 June 2022; version 1.8) [85].

All of the proposed correlation functions were fitted by Levenberg–Marquardt algorithm and visualized using Origin software (OriginLab Corporation, Northampton, MA, USA) [86]. The complexes with X⋯H (X = N, O, F, Cl, Br or I; H are protons of methyl groups) contacts shorter than the sum of Bondi’s van der Waals radii of corresponding atoms are marked in red in the Supplementary Materials (Figure S1 and Tables S1 and S2).

## 4. Conclusions

The summary and the main conclusion of this work are rather concise. For a homologous series of 145 halogen-bonded complexes with the general formula R–X⋯OPMe_3_ (X = F, Cl, Br, and I), we showed that the XB strength/energy correlate well with the distance between ED and ESP minima along the X⋯O bond path, Δ*d* (see Equation (2); Figure 4b). The maximum Δ*d* value (for weakest XBs) as well as the exponent of the fall of the Δ*d* dependence on *G*(*r*_BCP_) are halogen-independent, whereas the limiting values for strongest XBs are halogen-dependent. One could expect that there is also a dependence on the type of electron-donating atom (here, oxygen), though this and other limits of applicability of the proposed correlations remain to be studied. Quite likely, the most robust conclusion one could make is that for a pair of homologous non-covalently bound complexes the one with larger Δ*d* is weaker. The numerical value of Δ*d* might appear to be useful in analysis of experimental high-resolution X-ray data on ED of halogen-bonded single crystals. We also propose Δ*d* as a new tool for routine QTAIM analysis of the energies of XBs.

## Figures and Tables

**Table 1:** Fitting parameters $a_{X}$, Δ*d*_0_ and *b* for Equation (2) and proportionality coefficient *k* between *G*(*r*_BCP_) and complexation energy Δ*E* (Equation (4) [69]) for the data sets plotted in Figure 4b.

Halogen Donor	$a_{X},\;Å$	Δ*d*_0_, Å	*b*, kJ/(mol⋅Å^3^)	*k*, Å^3^
F	0.09	0.47	240	0.18
Cl	0.15	0.47	240	0.47
Br	0.18	0.47	240	0.57
I	0.19	0.47	240	0.74

## Data Availability

All data are available within the article or Supplementary Materials.

## Supplementary Materials

The following supporting information can be downloaded at: https://www.mdpi.com/article/10.3390/molecules27154848/s1. Figure S1: optimized structures of R–X⋯OPMe_3_ complexes; Table S1: normalized *R*_norm_ and absolute *R*(X⋯O) distances, valence bond angles *α*(R–X⋯O) and *β*(X⋯O–P), changes of ^31^P NMR chemical shifts upon complexation Δ*δ*^31^P, and complexation energies Δ*H* and Δ*G*; Table S2: minima positions of ED and ESP, *d*(*ED*_min_) and *d*(*ESP*_min_), along the XB path and distance between them Δ*d*, and QTAIM parameters at XB critical point of type (3; −1) (*ρ*(*r*_BCP_), ∇^2^*ρ*(*r*_BCP_), *G*(*r*_BCP_), *V*(*r*_BCP_), *K*(*r*_BCP_)); Figure S2: ED and ESP profiles along the XB path for weak, medium, and strong R–Cl⋯OPMe_3_ complexes; Figure S3: optimized structures of Me_3_POX^+^ (X = F, Cl, Br, and I) complexes; Figure S4: Δ*H* as a function of *G*(*r*_BCP_); Figures S5–S7: *d*(*ESP*_min_), *d*(*ED*_min_), Δ*d*, and *R*_norm_ as functions of electron density *ρ*(*r*_BCP_), Laplacian of electron density ∇^2^*ρ*(*r*_BCP_), and total electron energy density *K*(*r*_BCP_) at XB critical point of type (3; –1); Figure S8: *V*(*r*_BCP_), *ρ*(*r*_BCP_), ∇^2^*ρ*(*r*_BCP_), and *ESP*(*r*_BCP_) as functions of *G*(*r*_BCP_); Figure S9: Δ*H*, *ρ*(*r*_BCP_), ∇^2^*ρ*(*r*_BCP_), and *ESP*(*r*_BCP_) as functions of Δ*d*; Figure S10: *R*_norm_ and *R*(X⋯O) as functions of *G*(*r*_BCP_); Figure S11: Δ*H* and *G*(*r*_BCP_) as functions of Δ*δ*^31^P.

## Sample Availability

Samples of the compounds are available from the authors.
