# Supplementary material for: The Distance between Minima of Electron Density and Electrostatic Potential as a Measure of Halogen Bond Strength
Source: Molecules. 2022 Jul 28;27(15):4848. doi: 10.3390/molecules27154848 (PMC9369751; doi:10.3390/molecules27154848)

Supplementary materials for

The distance between minima of electron density and electrostatic potential as a measure of halogen bond strength

Edem R. Chakalov,[1] Elena Yu. Tupikina,[1] Daniil M. Ivanov,[1,]*

Ekaterina V. Bartashevich,[2] Peter M. Tolstoy[1,]*

[1] *Institute of Chemistry, St. Petersburg State University, 198504 St. Petersburg, Russia;*

[2] *Chemistry Department, South Ural State University, 454080 Chelyabinsk, Russia*

**corresponding authors: d.m.ivanov@spbu.ru, peter.tolstoy@spbu.ru*

Contents **Page**

Figure S1. Calculated optimized structures of R–X···$OPMe_3$ complexes. **2**

Table S1. Calculated halogen bond lengths and angles, ^{31}P NMR chemical shifts, complexation enthalpies and Gibbs energies for R–X···$OPMe_3$ complexes. **10**

Table S2. Calculated halogen bond QTAIM parameters at X···O bond critical point. **13**

Figure S2. ED and ESP profiles along the X···O bond path for weak, medium and strong R–Cl···$OPMe_3$ complexes. **16**

Figure S3. Optimized structures of Me_3POX^+ complexes with $G(r_{BCP})$ and Δd values for the X···O bonds. **17**

Figure S4. Complexation enthalpy as a function of local electron kinetic energy density at bond critical point. **18**

Figure S5. Distances from oxygen atom to minima of electron density and molecular electrostatic potential along the X···O bond path, the distance between them and normalized X···O distances as a function of electron density at bond critical point. **19**

Figure S6. Distances from oxygen atom to minima of electron density and molecular electrostatic potential along the X···O bond path, the distance between them and normalized X···O distances as a function of Laplacian of electron density at bond critical point. **20**

Figure S7. Distances from oxygen atom to minima of electron density and molecular electrostatic potential along the X···O bond path, the distance between them and normalized X···O distances as a function of total electron energy density at bond critical point. **21**

Figure S8. Local electron potential energy density, electron density, Laplacian of electron density and molecular electrostatic potential as a function of local electron kinetic energy density at X···O bond critical point. **22**

Figure S9. Complexation enthalpy, electron density, Laplacian of electron density and molecular electrostatic potential at X···O bond critical point as a function of distance between ED and ESP minima along the bond path. **23**

Figure S10. Absolute and normalized values of X···O distances as a function of local electron kinetic energy density at bond critical point. **24**

Figure S11. Complexation enthalpy and local electron kinetic energy density at X···O bond critical point as a function of the change of the ^{31}P NMR chemical shift upon complexation. **25**

Figure S1. Calculated (B3LYP/jorge-ATZP, vacuum) optimized structures of 145 intermolecular 1:1 complexes formed by Me_3PO with halogen-donating molecules. The complexes with additional Y···H (Y = N, O, F, Cl, Br or I; H are protons of methyl groups) contacts shorter than the sum of Bondi's van der Waals radii of corresponding atoms are marked in red color.

**Halogens**		
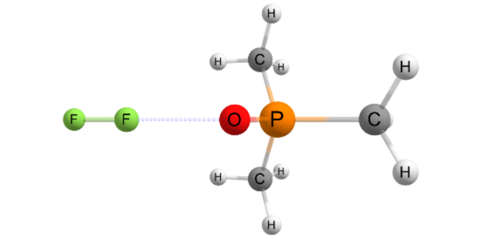	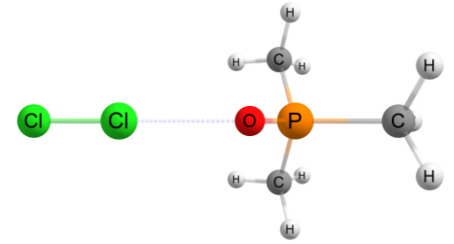	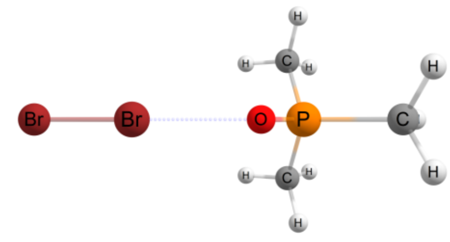
**F_2 [1]**	**Cl_2 [2]**	**Br_2 [3]**
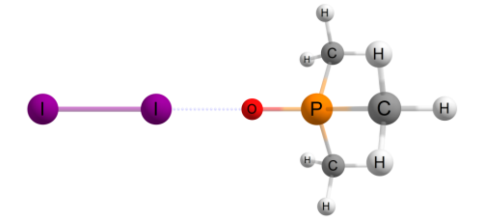	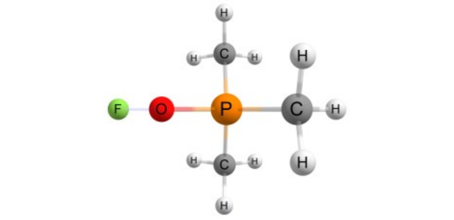	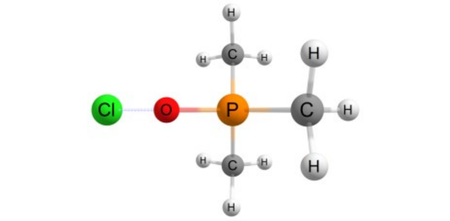
**I_2 [4]**	**F^+ [5]**	**Cl^+ [6]**
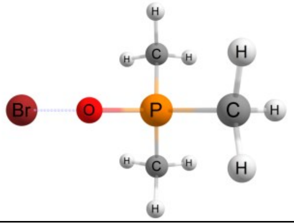	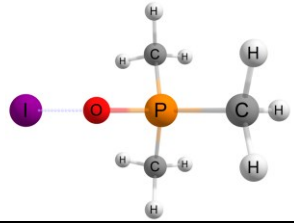	
**Br^+ [7]**	**I^+ [8]**	

**Interhalides**		
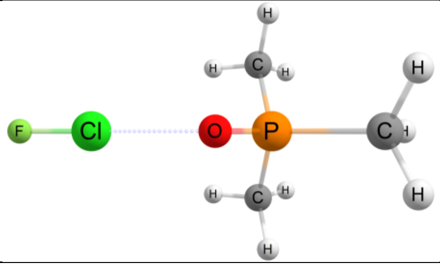	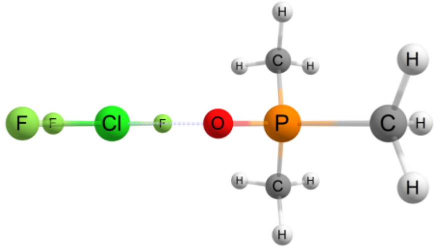	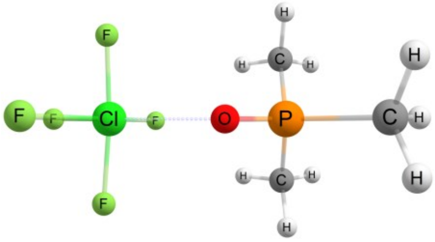
**ClF [9]**	**ClF_3 [10]**	**ClF_5 [11]**
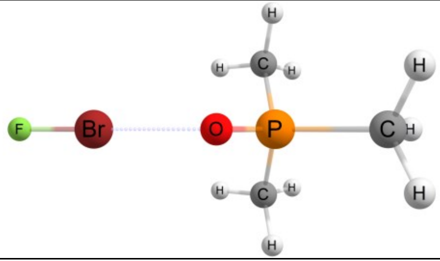	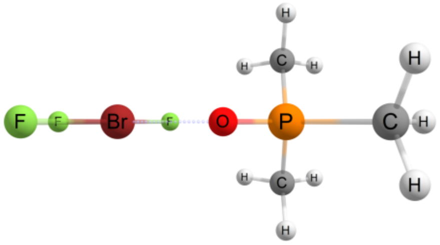	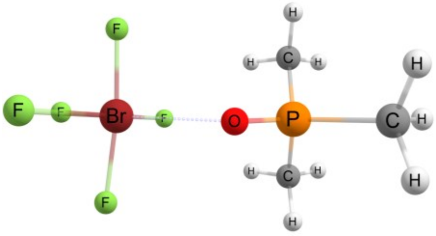
**BrF [12]**	**BrF_3 [13]**	**BrF_5 [14]**
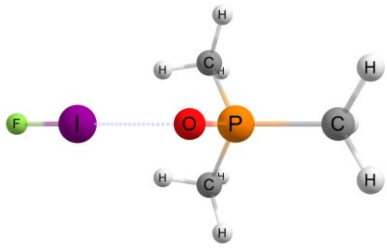	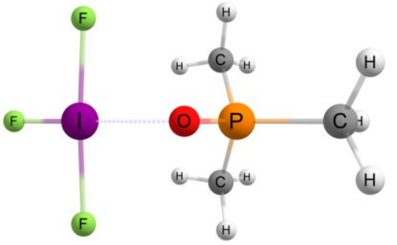	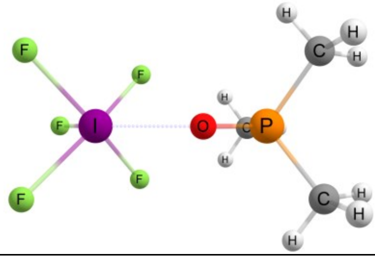
**IF [15]**	**IF_3 [16]**	**IF_5 [17]**

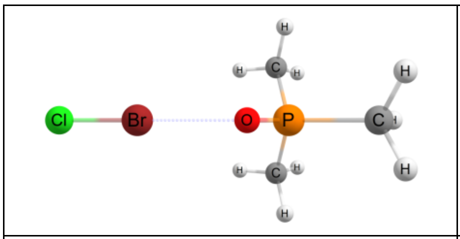	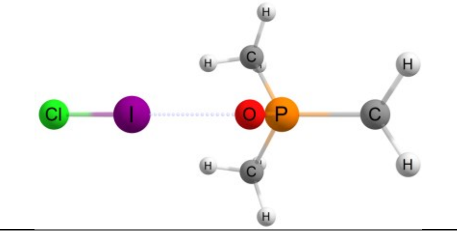	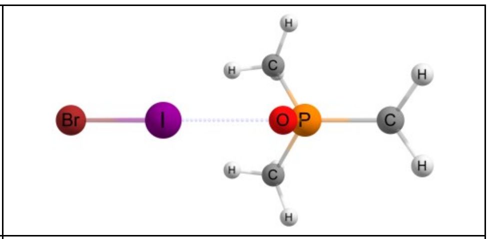
**BrCl [18]**	**ICl [19]**	**IBr [20]**

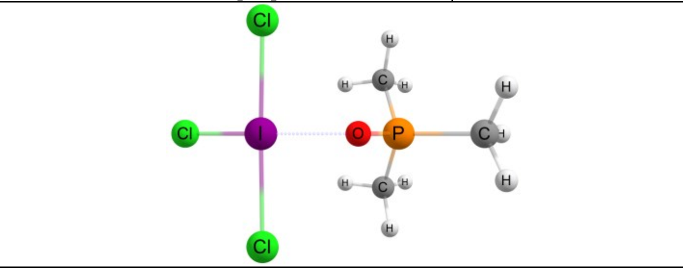	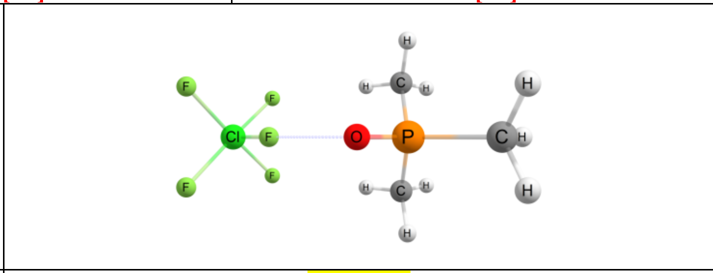
**ICl_3 [21]**	**ClF_5 [22]**

**Oxohalides**		
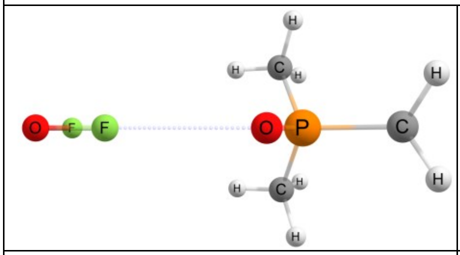	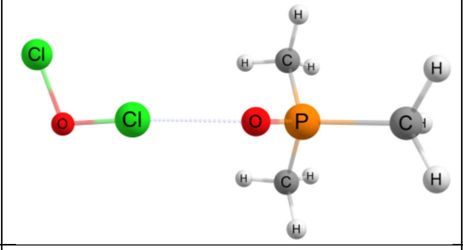	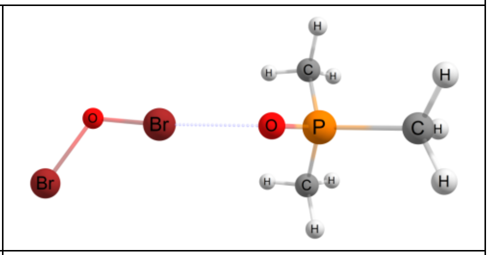
**OF_2 [23]**	**Cl_2O [24]**	**Br_2O [25]**
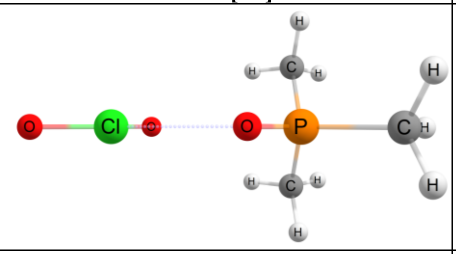	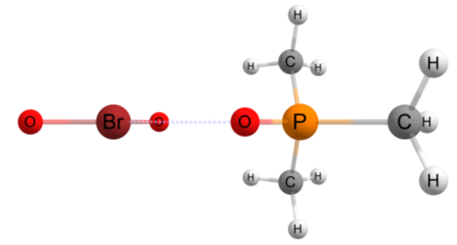	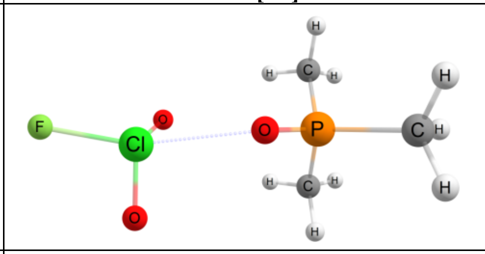
**ClO_2 [26]**	**BrO_2 [27]**	**ClO_2F [28]**
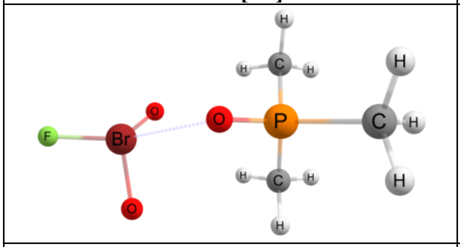	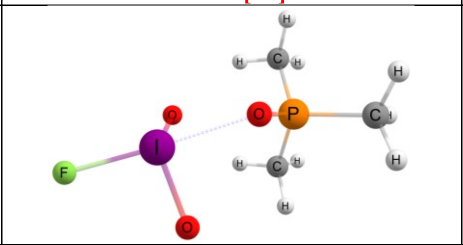	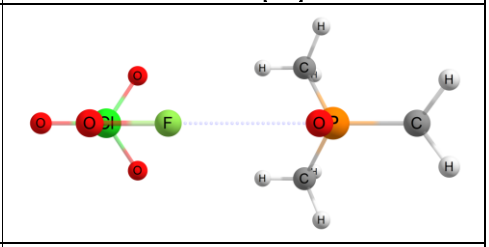
**BrO_2F [29]**	**IO_2F [30]**	**ClO_3OF [31]**
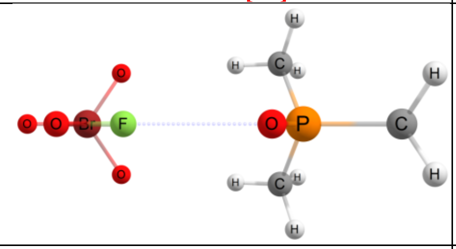	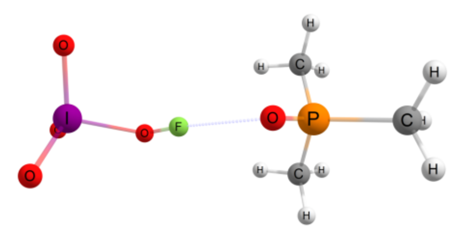	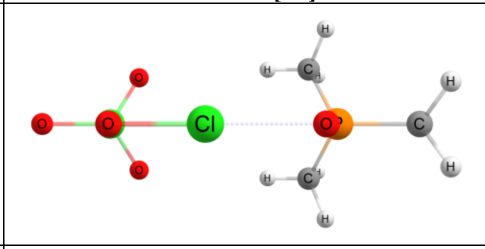
**BrO_3OF [32]**	**IO_3OF [33]**	**ClO_3OCl [34]**

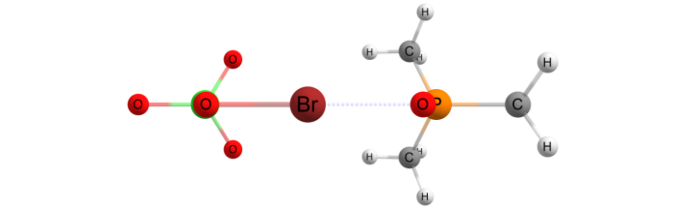	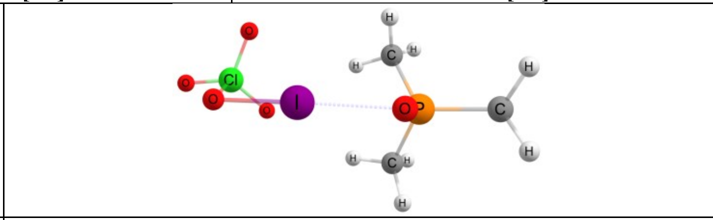
**ClO_3OBr [35]**	**ClO_3OI [36]**

Pseudohalides		
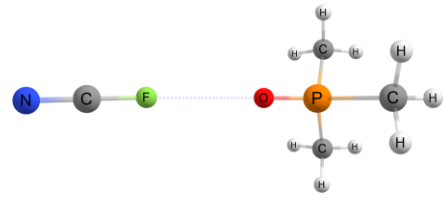	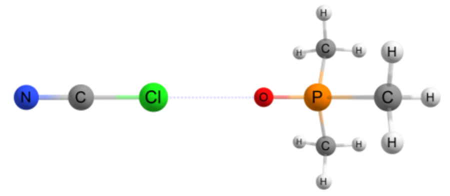	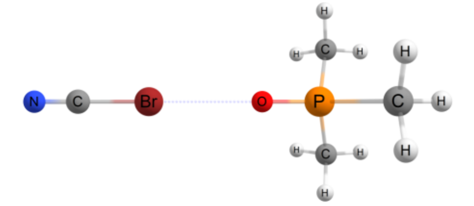
**FCN [37]**	**ClCN [38]**	**BrCN [39]**
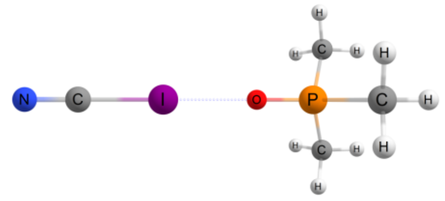	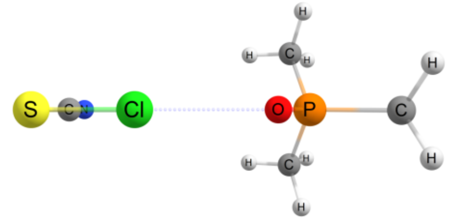	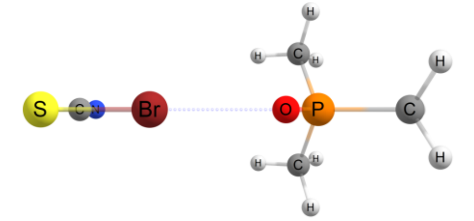
**ICN [40]**	**ClSCN [41]**	**BrSCN [42]**
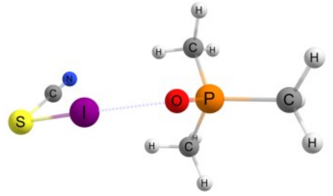	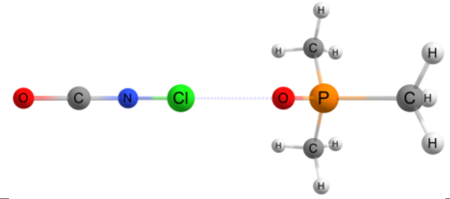	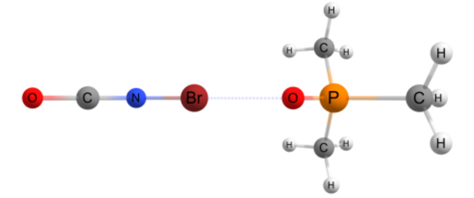
**ISCN [43]**	**ClNCO [44]**	**BrNCO [45]**
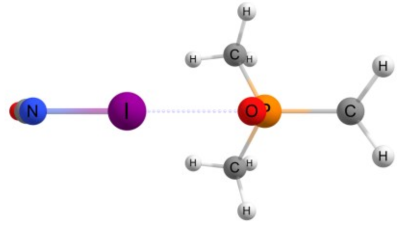	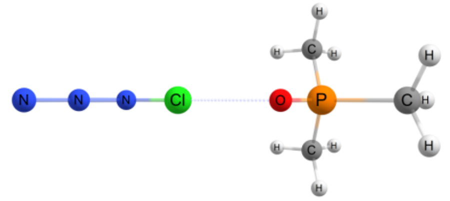	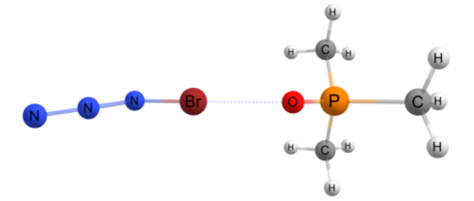
**INCO [46]**	**ClN_3 [47]**	**BrN_3 [48]**
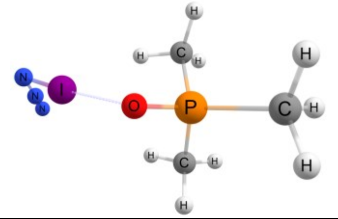		
**IN_3 [49]**		

Halogenated methanes and their derivatives		
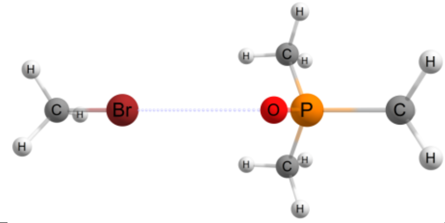	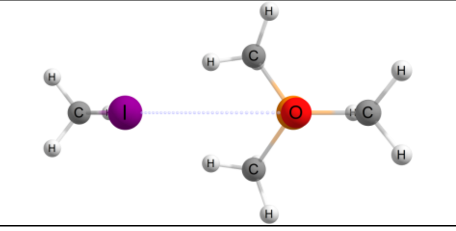	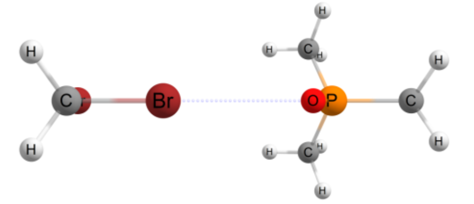
**CH_3Br [50]**	**CH_3I [51]**	**CH_2Br_2 [52]**
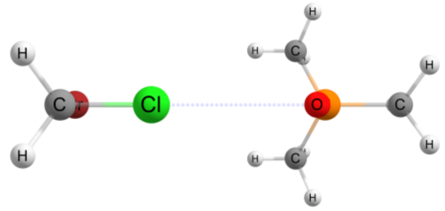	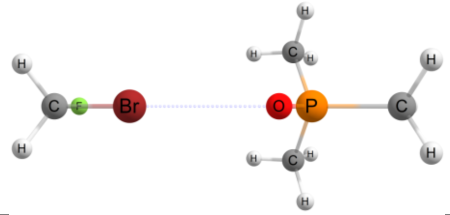	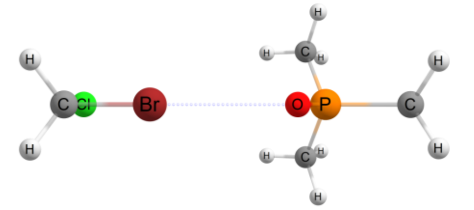
**CH_2ClBr [53]**	**CH_2BrF [54]**	**CH_2BrCl [55]**

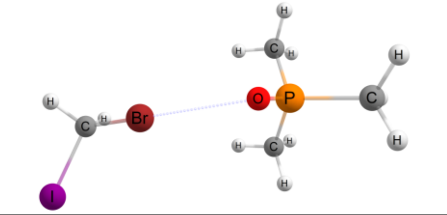	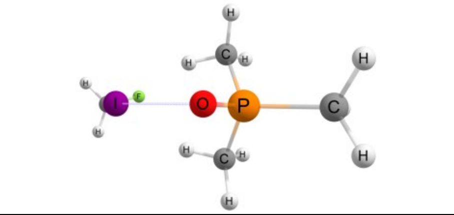	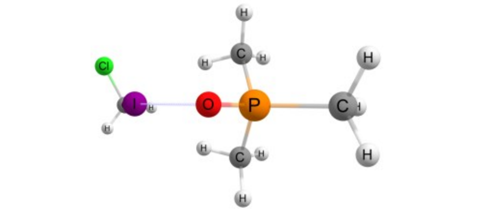
CH_2BrI [56]	CH_2IF [57]	CH_2ICl [58]
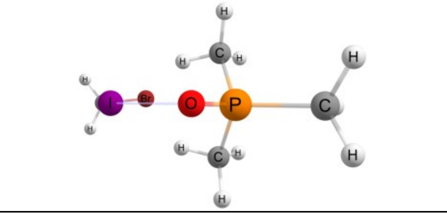	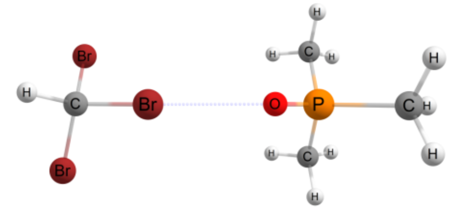	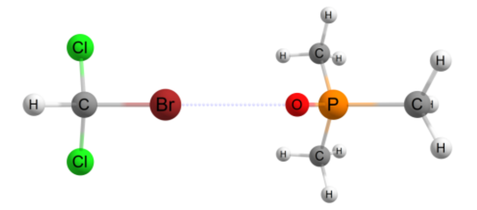
CH_2IBr [59]	$CHBr_3$ [60]	$CHBrCl_2$ [61]
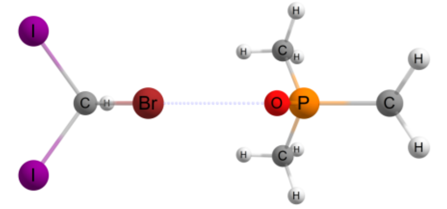	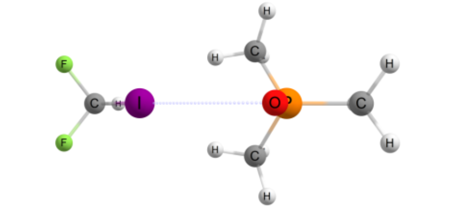	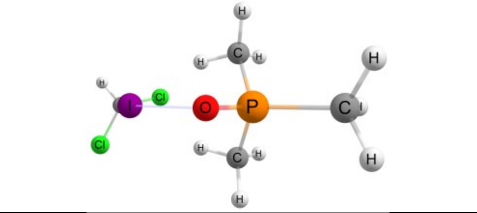
$CHBrI_2$ [62]	$CHIF_2$ [63]	$CHICl_2$ [64]
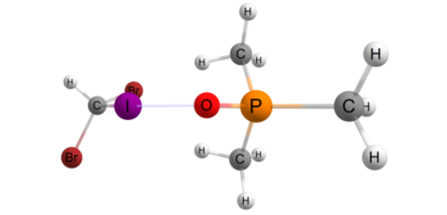	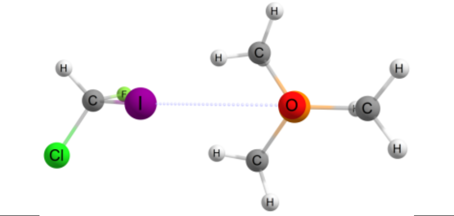	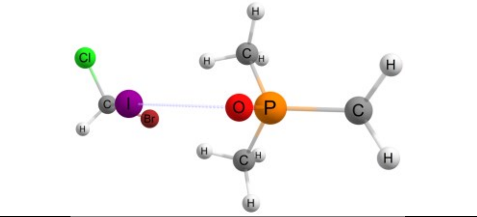
$CHIBr_2$ [65]	$CHIClF$ [66]	$CHIClBr$ [67]
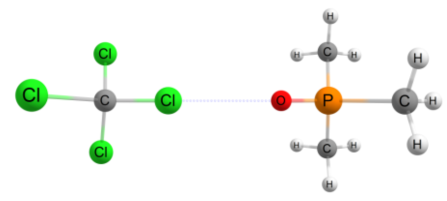	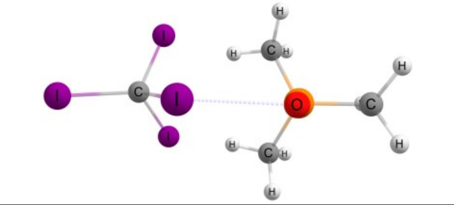	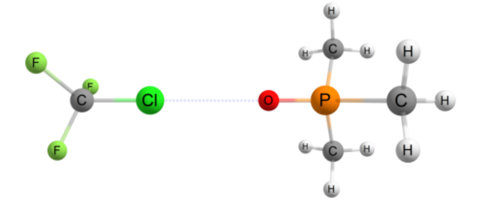
CCl_4 [68]	CI_4 [69]	CF_3Cl [70]
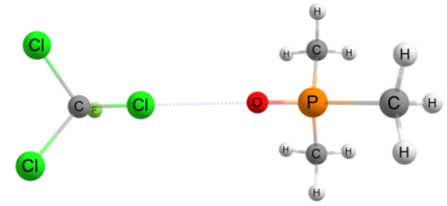	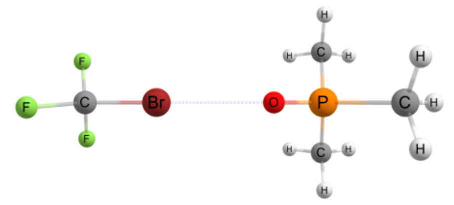	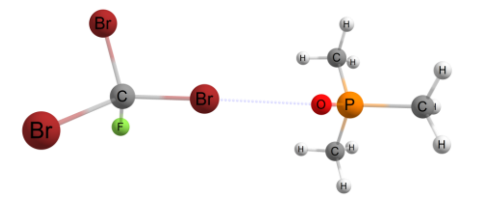
CCl_3F [71]	CF_3Br [72]	CBr_3F [73]
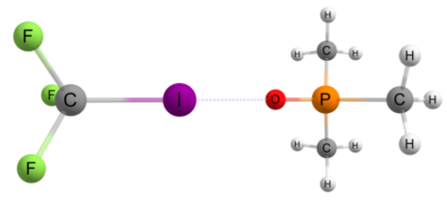	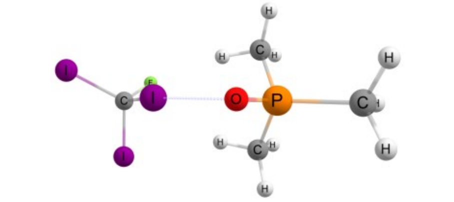	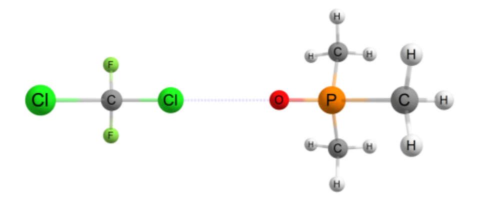
CF_3I [74]	CI_3F [75]	CCl_2F_2 [76]
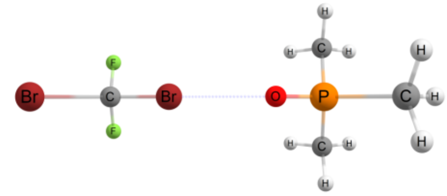	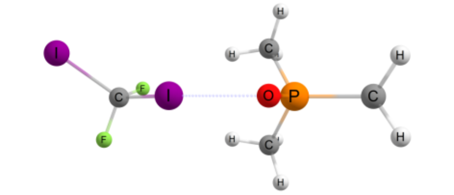	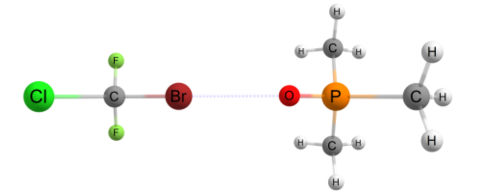
CBr_2F_2 [77]	CI_2F_2 [78]	$CBrClF_2$ [79]

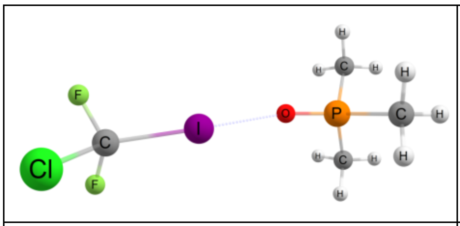	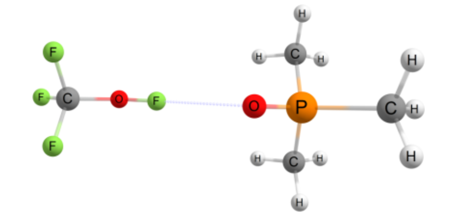	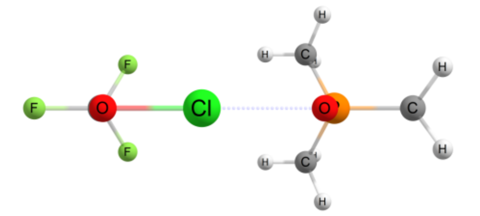
$CIClF_2$ [80]	CF_3OF [81]	CF_3OCl [82]
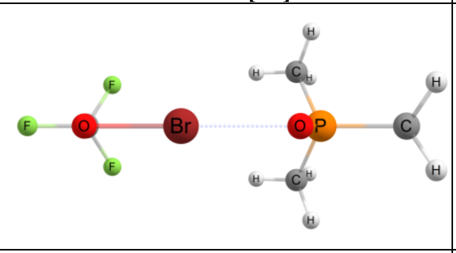	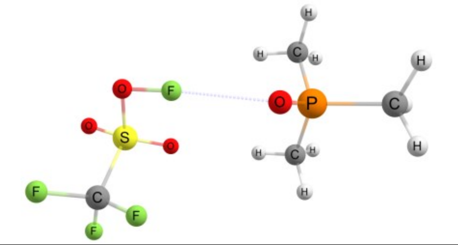	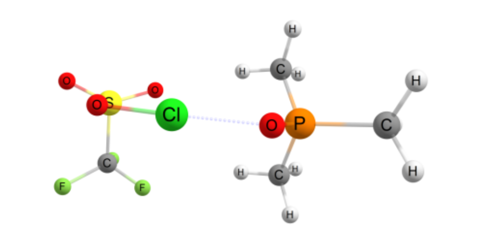
CF_3OBr [83]	CF_3SO_2OF [84]	CF_3SO_2OCl [85]
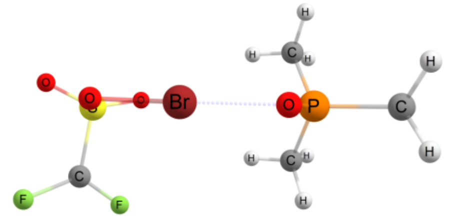		
CF_3SO_2OBr [86]		

Halogenated ethylene, halogenated acetylene and their derivatives		
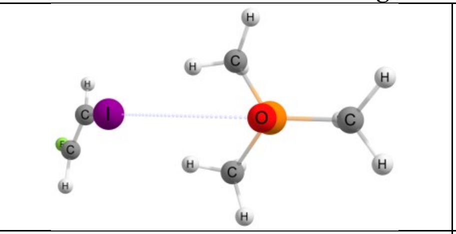	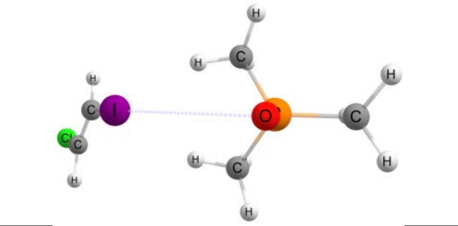	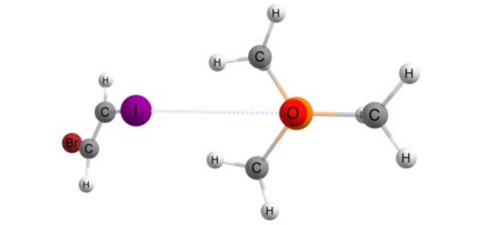
C_2H_2IF [87]	C_2H_2ICl [88]	C_2H_2IBr [89]
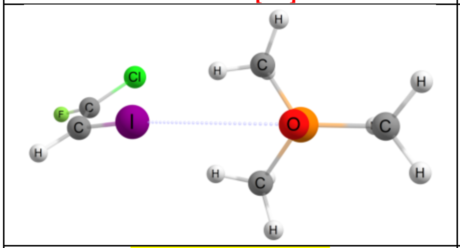	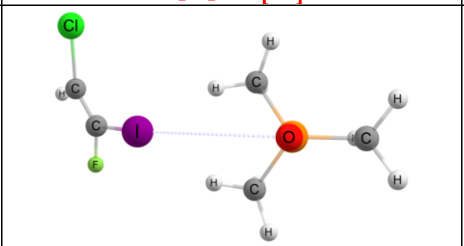	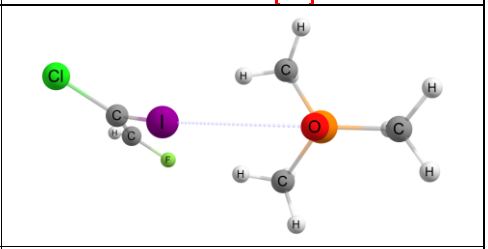
CI(H)C(F)Cl [90]	CI(F)C(H)Cl [91]	CI(Cl)C(H)F [92]
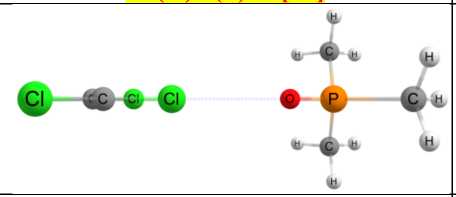	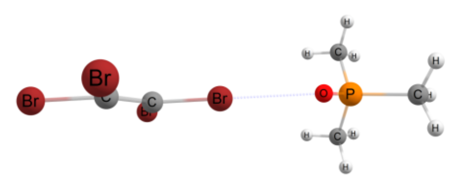	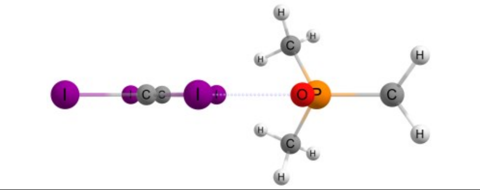
C_2Cl_4 [93]	C_2Br_4 [94]	C_2I_4 [95]
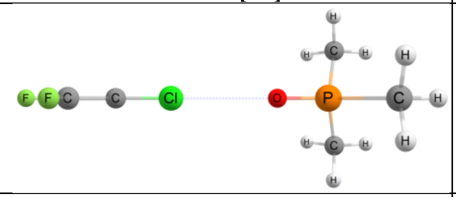	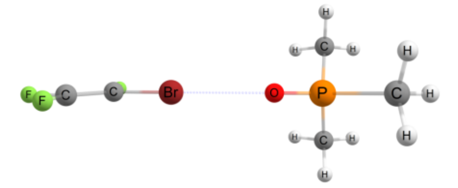	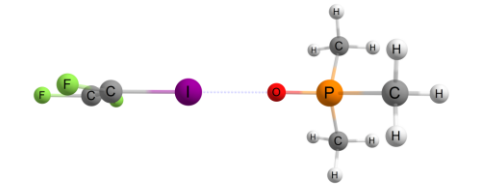
C_2F_3Cl [96]	C_2F_3Br [97]	C_2F_3I [98]

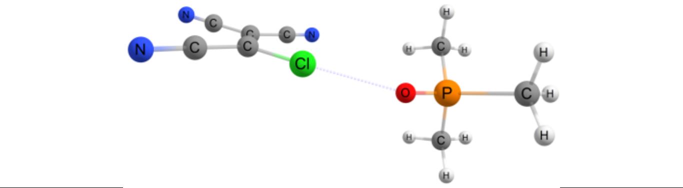	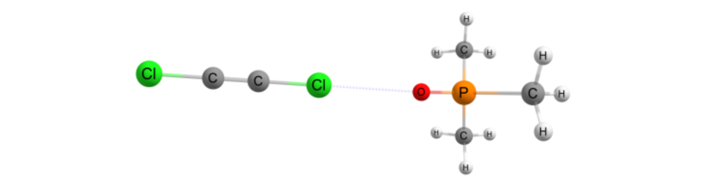
$C_2(CN)_3Cl$ [99]	C_2Cl_2 [100]

Halogenated benzenes

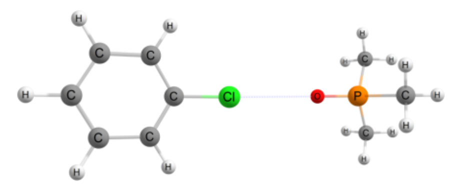	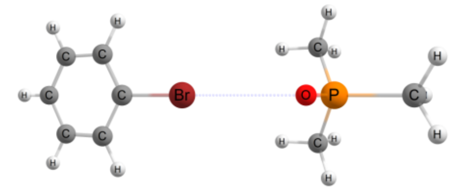	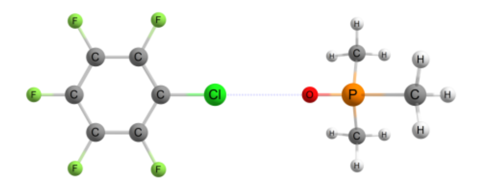
C_6H_5Cl [101]	C_6H_5Br [102]	C_6F_5Cl [103]
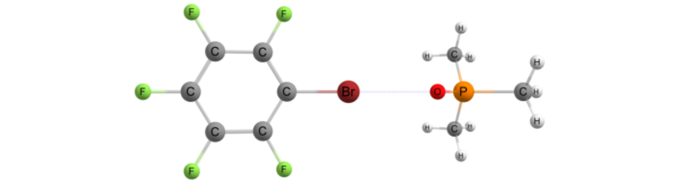	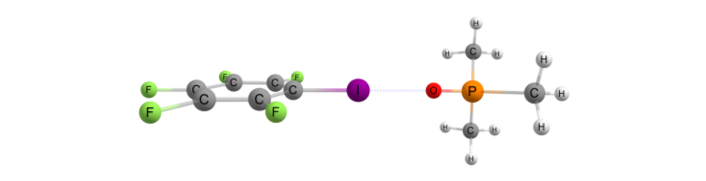	
C_6F_5Br [104]	C_6F_5I [105]	

Phosgene and its derivatives

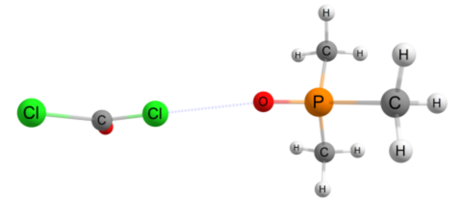	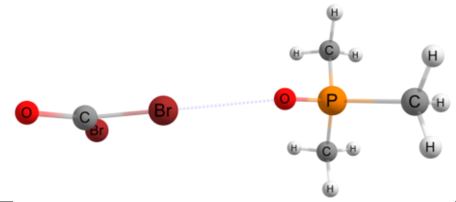	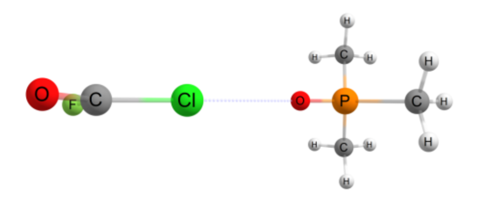
$COCl_2$ [106]	$COBr_2$ [107]	COClF [108]
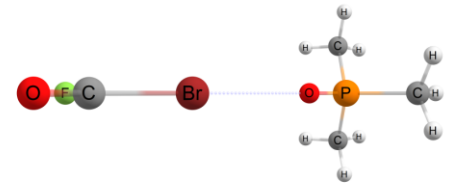	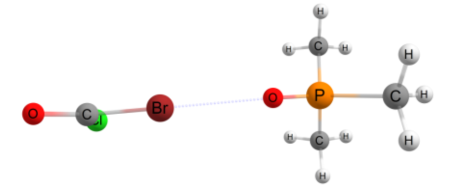	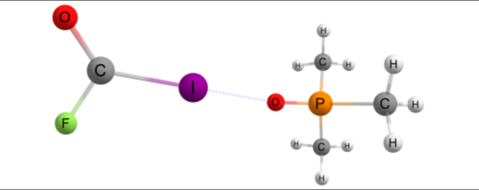
COBrF [109]	COBrCl [110]	COIF [111]

Thionyl- and sulfurylhalides

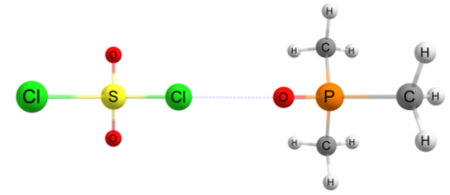	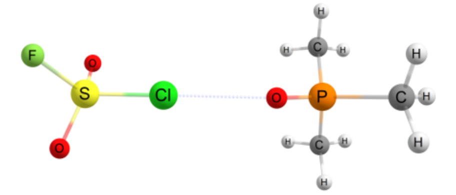	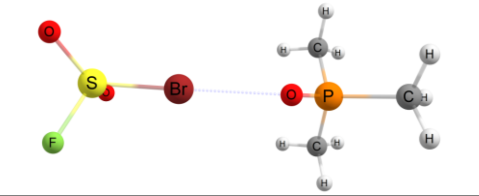
SO_2Cl_2 [112]	SO_2ClF [113]	SO_2BrF [114]
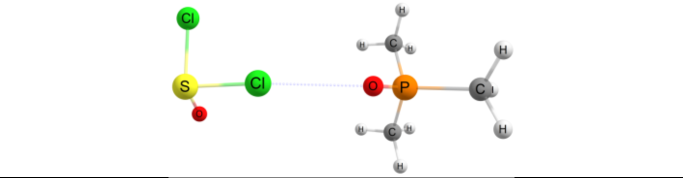	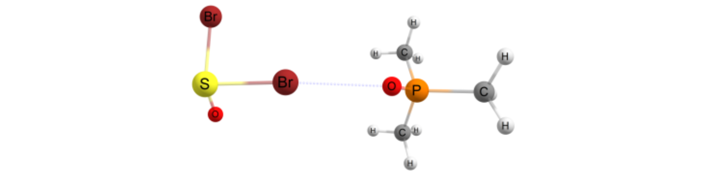	
$SOCl_2$ [115]	$SOBr_2$ [116]	

Sulfur halides and sulfur hypohalites		
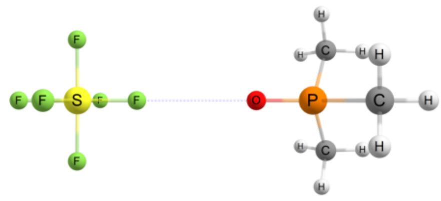	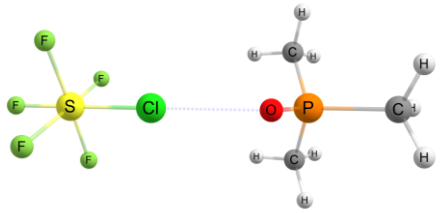	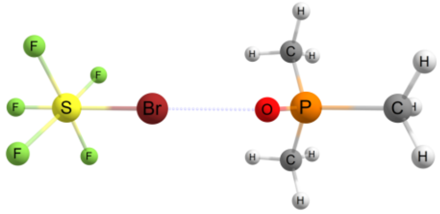
SF_6 [117]	SF_5Cl [118]	SF_5Br [119]
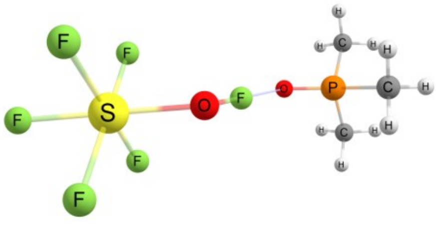	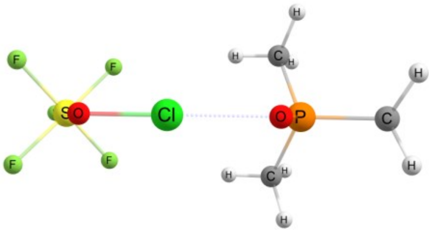	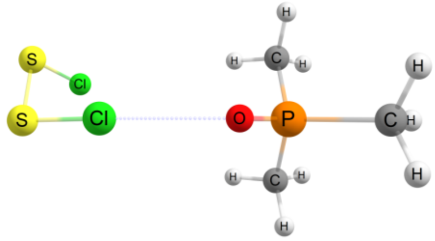
SF_5OF [120]	SF_5OCl [121]	S_2Cl_2 [122]
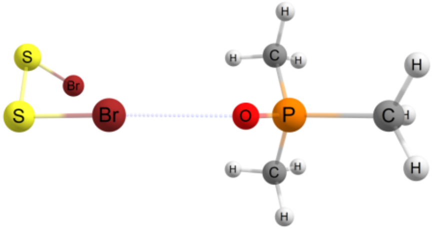	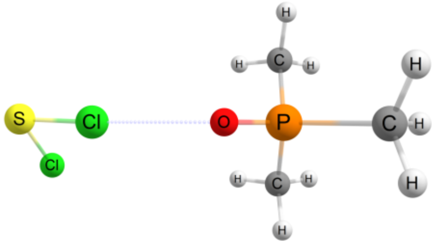	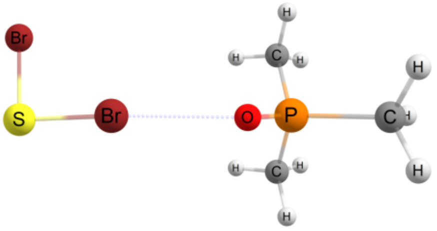
S_2Br_2 [123]	SCl_2 [124]	SBr_2 [125]

Halogenated nitrogen-containing inorganic compounds		
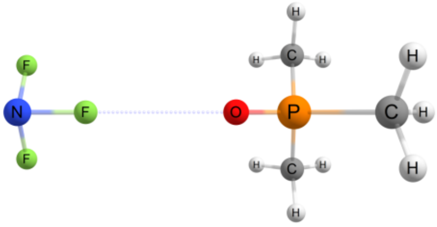	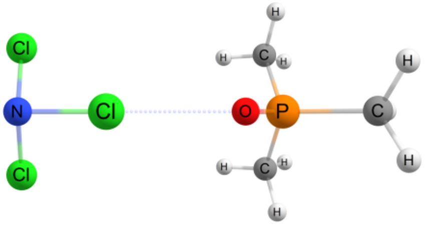	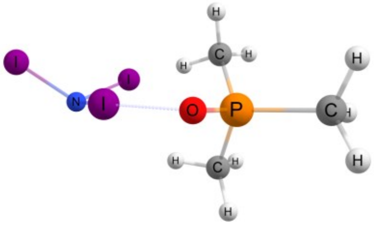
NF_3 [126]	NCl_3 [127]	NI_3 [128]
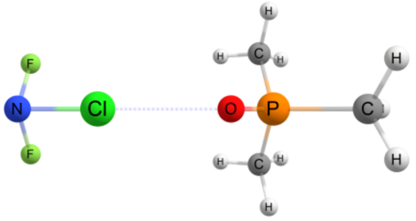	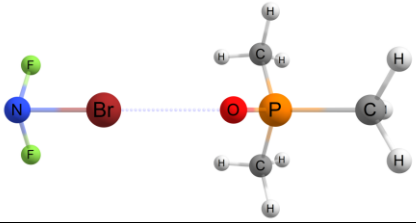	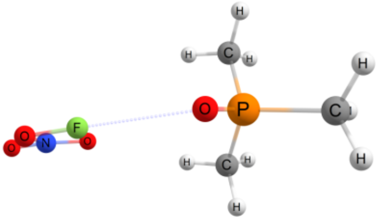
NF_2Cl [129]	NF_2Br [130]	NO_2OF [131]
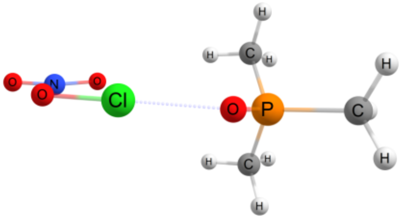	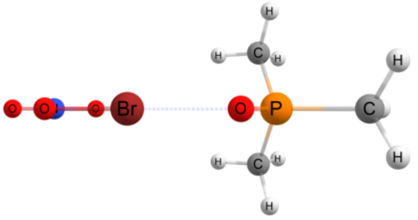	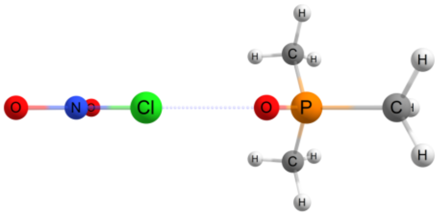
NO_2OCl [132]	NO_2OBr [133]	NO_2Cl [134]
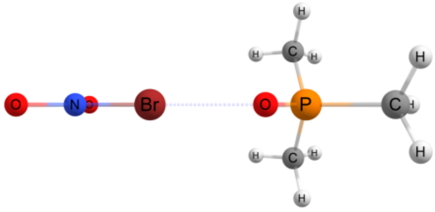	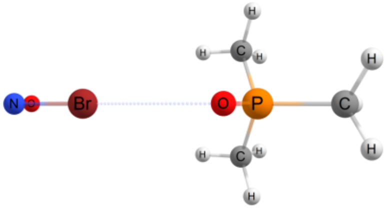	
NO_2Br [135]	NOBr [136]	

Assorted organic compounds		
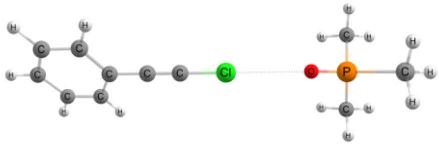	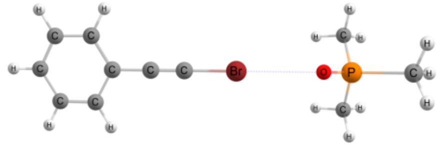	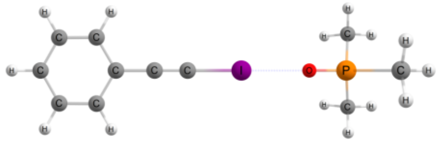
**$C_6H_5C_2Cl$ [137]**	**$C_6H_5C_2Br$ [138]**	**$C_6H_5C_2I$ [139]**
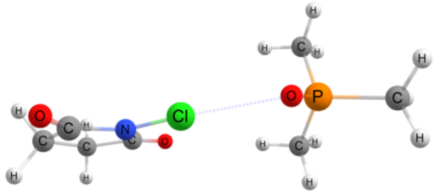	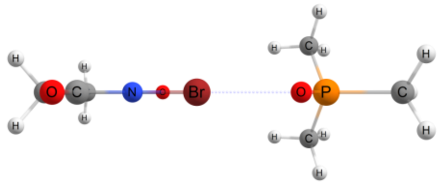	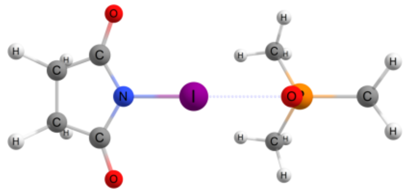
**N-Chlorosuccinimide [140]**	**N-Bromosuccinimide [141]**	**N-Iodosuccinimide [142]**
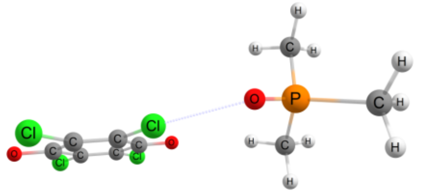	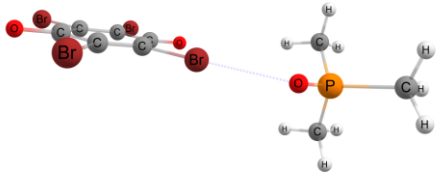	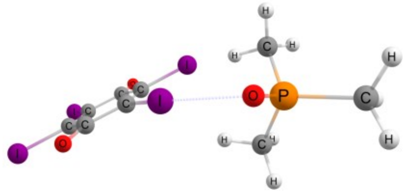
**Tetrachloro-1,4-benzoquinone [143]**	**Tetrabromo-1,4-benzoquinone [144]**	**Tetraiodo-1,4-benzoquinone [145]**

Table S1. Results of DFT calculation (B3LYP/jorge-ATZP, vacuum) of 145 intermolecular 1:1 complexes formed by Me_3PO with halogen-donating molecules: optimized halogen bond lengths R and normalized (by the sum of van der Waals radii of oxygen and halogen) halogen bond lengths R_{norm}, valence bond angles α(R–X···O) and β(X···O–P), ^{31}P NMR chemical shifts $\Delta\delta^{31}$P, complexation enthalpies ΔH and Gibbs energies ΔG. The complexes with additional Y···H (Y = N, O, F, Cl, Br or I; H are protons of methyl groups) contacts shorter than the sum of Bondi's van der Waals radii of corresponding atoms are marked in red color. In ambiguous cases the halogen atoms which participate in halogen bond formation are highlighted in bold.

**№**	**Halogen donor**	***R*, Å**	**R_{norm}**	**α(R–X···O), °**	**β(X···O–P), °**	**$\Delta\delta^{31}$P, ppm**	**ΔH, kJ/mol**	**ΔG, kJ/mol**
				**Halogens**				
**1**	F_2	2.259	0.755	179.5	103.2	12.25	-9.32	22.75
**2**	Cl_2	2.514	0.769	179.6	116.5	13.59	-17.74	14.43
**3**	Br_2	2.528	0.750	179.8	119.0	17.48	-27.38	6.04
**4**	I_2	2.229	0.637	180.0	179.9	1.01	-136.59	-103.20
**5**	F^+	1.431	0.479	–	106.3	120.78	-1348.96	-1315.91
**6**	Cl^+	1.718	0.525	–	116.7	115.44	-788.55	-755.61
**7**	Br^+	1.857	0.551	–	119.7	110.09	-655.60	-622.88
**8**	I^+	1.951	0.557	–	113.6	106.16	-722.03	-686.70
				**Interhalides**				
**9**	$\mathbf{Cl}F$	2.287	0.699	180.0	115.0	22.91	-40.17	-1.93
**10**	$\mathbf{Cl}F_3$	2.264	0.692	179.3	122.9	27.70	-52.88	-9.21
**11**	$\mathbf{Cl}F_5$	2.494	0.763	177.5	125.7	18.59	-35.59	0.14
**12**	$\mathbf{Br}F$	2.331	0.692	180.0	118.3	25.58	-55.32	-16.50
**13**	$\mathbf{Br}F_3$	2.298	0.682	177.5	124.6	30.67	-70.44	-26.30
**14**	$\mathbf{Br}F_5$	2.525	0.749	171.4	129.4	21.03	-49.88	-14.42
**15**	$\mathbf{I}F$	2.252	0.644	172.8	105.8	49.39	-186.49	-135.94
**16**	$\mathbf{I}F_3$	2.171	0.620	178.2	109.8	48.87	-178.16	-128.81
**17**	$\mathbf{I}F_5$	2.347	0.671	151.7	137.5	24.12	-107.67	-64.58
**18**	$\mathbf{Br}Cl$	2.469	0.733	179.8	119.1	19.50	-33.32	2.81
**19**	$\mathbf{I}Cl$	2.309	0.660	172.5	104.8	43.77	-154.34	-104.66
**20**	$\mathbf{I}Br$	2.321	0.663	173.7	104.6	42.89	-149.38	-100.92
**21**	$\mathbf{I}Cl_3$	2.207	0.631	174.5	111.8	46.38	-158.89	-108.35
**22**	$Cl\mathbf{F}_5$	2.480	0.829	175.8	109.4	8.00	-5.31	24.19
				**Oxohalides**				
**23**	OF_2	2.840	0.950	178.7	114.3	0.94	2.53	20.98
**24**	Cl_2O	2.450	0.749	176.2	117.1	15.33	-21.55	14.37
**25**	Br_2O	2.446	0.726	176.0	119.4	19.16	-34.60	3.90
**26**	ClO_2	2.817	0.862	154.9	124.4	7.76	-20.10	14.04
**27**	BrO_2	2.721	0.807	161.4	124.1	12.40	-34.13	5.25
**28**	$\mathbf{Cl}O_2F$	2.619	0.801	151.5	128.8	11.72	-29.79	3.12
**29**	$\mathbf{Br}O_2F$	2.474	0.734	159.3	123.5	22.06	-52.11	-9.92
**30**	$\mathbf{I}O_2F$	2.146	0.613	176.6	121.0	39.97	-191.53	-137.80
**31**	$ClO_3O\mathbf{F}$	2.722	0.910	176.7	119.5	2.53	0.96	26.74
**32**	$BrO_3O\mathbf{F}$	2.661	0.890	173.8	119.7	3.17	-1.27	28.57
**33**	$IO_3O\mathbf{F}$	2.512	0.840	173.1	101.7	3.79	-12.70	22.49
**34**	$ClO_3O\mathbf{Cl}$	2.249	0.688	178.1	114.5	27.49	-35.81	5.45
**35**	$ClO_3O\mathbf{Br}$	2.238	0.664	178.1	116.8	35.63	-60.43	-18.07
**36**	$ClO_3O\mathbf{I}$	2.175	0.622	175.2	103.1	45.90	-192.85	-143.24
				**Pseudohalides**				
**37**	FCN	2.860	0.957	176.4	179.7	1.86	-4.05	8.61
**38**	ClCN	2.773	0.848	180.0	179.9	4.21	-17.92	4.53
**39**	BrCN	2.729	0.810	179.9	161.1	5.16	-24.30	-5.95
**40**	ICN	2.328	0.665	180.0	180.0	2.40	-99.07	-63.81
**41**	ClSCN	2.695	0.824	179.9	123.1	7.87	-11.58	18.12
**42**	BrSCN	2.587	0.768	179.9	122.0	14.71	-24.53	9.50
**43**	ISCN	2.318	0.662	173.5	106.5	37.70	-138.97	-86.99
**44**	ClNCO	2.594	0.793	178.0	120.7	8.06	-14.42	16.23
**45**	BrNCO	2.505	0.743	178.1	121.7	14.72	-29.61	4.19
**46**	INCO	2.269	0.648	177.0	105.5	30.90	-140.43	-94.88
**47**	ClN_3	2.647	0.809	177.0	119.6	7.14	-10.71	18.68
**48**	BrN_3	2.556	0.759	177.5	121.5	15.83	-23.93	7.77
**49**	IN_3	2.333	0.666	175.9	108.2	40.48	-135.69	-81.95

**Halogenated methanes and their derivatives**								
**50**	CH_3Br	3.258	0.967	176.3	111.5	1.99	2.76	23.34
**51**	CH_3I	3.429	0.980	162.8	83.7	8.48	-87.51	-43.93
**52**	CH_2Br_2	3.005	0.892	178.4	121.5	3.30	-3.59	23.39
**53**	CH_2ClBr	3.155	0.965	176.2	112.8	1.42	1.11	25.26
**54**	CH_2BrF	3.093	0.918	176.0	118.9	3.69	-1.56	24.65
**55**	CH_2BrCl	3.018	0.896	178.0	121.3	3.36	-3.10	23.61
**56**	CH_2BrI	2.962	0.879	174.1	112.8	4.68	-8.95	24.26
**57**	CH_2IF	3.375	0.964	160.9	84.7	9.61	-90.49	-47.50
**58**	CH_2ICl	3.330	0.952	164.7	85.7	9.70	-87.07	-40.96
**59**	CH_2IBr	3.333	0.952	161.7	85.4	9.69	-90.48	-48.11
**60**	$CHBr_3$	2.878	0.854	179.5	128.8	3.44	-8.13	19.56
**61**	$CHBrCl_2$	2.892	0.858	179.7	128.3	6.34	-7.60	19.42
**62**	$CHBrI_2$	2.830	0.840	173.1	109.6	6.15	-19.99	15.58
**63**	$CHIF_2$	3.285	0.939	167.3	86.3	10.27	-89.36	-47.78
**64**	$CHICl_2$	3.250	0.929	163.4	87.1	11.81	-87.14	-41.56
**65**	$CHIBr_2$	2.617	0.748	173.0	99.6	24.49	-91.40	-43.16
**66**	CHIClF	3.267	0.934	162.7	86.7	11.08	-90.79	-45.63
**67**	CHIClBr	3.247	0.928	162.5	87.1	12.33	-90.49	-44.31
**68**	CCl_4	2.880	0.881	179.6	144.6	3.09	-4.63	22.03
**69**	CI_4	2.507	0.716	147.6	91.3	13.68	-225.91	-169.64
**70**	CF_3Cl	2.919	0.893	179.9	163.3	0.61	-5.20	18.24
**71**	CCl_3F	2.895	0.885	178.6	149.8	2.31	-4.84	19.33
**72**	CF_3Br	2.857	0.848	179.7	143.7	4.56	-11.14	16.54
**73**	CBr_3F	2.807	0.833	178.0	133.7	6.66	-11.70	16.11
**74**	CF_3I	2.348	0.671	180.0	179.7	2.50	-74.10	-44.13
**75**	CI_3F	2.515	0.719	164.0	100.3	27.79	-135.69	-85.15
**76**	CCl_2F_2	2.907	0.889	179.0	157.4	1.11	-4.98	17.08
**77**	CBr_2F_2	2.833	0.841	178.0	136.7	5.96	-11.31	16.55
**78**	CI_2F_2	2.530	0.723	170.3	101.1	26.05	-114.78	-64.60
**79**	$CBrClF_2$	2.837	0.842	178.5	138.6	4.80	-11.15	17.14
**80**	$CIClF_2$	2.345	0.670	177.2	168.7	2.31	-73.69	-41.35
**81**	CF_3OF	2.770	0.927	179.7	111.5	0.95	2.33	23.87
**82**	CF_3OCl	2.320	0.709	178.2	116.0	21.00	-32.33	5.41
**83**	CF_3OBr	2.311	0.686	178.0	118.4	27.41	-53.10	-14.08
**84**	CF_3SO_2OF	2.601	0.870	176.5	111.6	4.52	-2.54	29.10
**85**	CF_3SO_2OCl	2.118	0.648	178.7	111.3	39.14	-49.66	-5.38
**86**	CF_3SO_2OBr	2.183	0.648	179.0	115.3	41.48	-73.18	-30.84
**Halogenated ethylene, halogenated acetylene and their derivatives**								
**87**	C_2H_2IF	3.470	0.991	152.1	83.7	7.48	-90.39	-48.46
**88**	C_2H_2ICl	3.464	0.990	152.0	83.9	8.50	-88.90	-47.06
**89**	C_2H_2IBr	3.452	0.986	152.6	84.1	9.03	-89.55	-47.23
**90**	CI(H)C(F)Cl	3.291	0.940	167.9	86.3	11.10	-86.20	-40.70
**91**	CI(F)C(H)Cl	3.307	0.945	159.4	86.6	9.24	-89.45	-44.86
**92**	CI(Cl)C(H)Cl	3.284	0.938	162.7	86.5	11.51	-86.34	-41.07
**93**	C_2Cl_4	2.943	0.900	179.5	152.5	2.16	-3.07	22.20
**94**	C_2Br_4	2.861	0.849	178.5	135.9	4.57	-9.48	17.53
**95**	C_2I_4	2.470	0.706	168.0	94.6	16.87	-204.58	-143.54
**96**	C_2F_3Cl	2.942	0.900	178.9	157.4	0.34	-4.44	19.70
**97**	C_2F_3Br	2.877	0.854	178.8	146.8	4.08	-10.71	15.23
**98**	C_2F_3I	2.373	0.678	179.1	178.4	0.46	-65.54	-33.23
**99**	$C_2(CN)_3Cl$	2.744	0.839	174.4	138.8	6.59	-18.37	12.28
**100**	C_2Cl_2	2.906	0.889	179.9	164.7	2.15	-6.97	14.92
**Halogenated benzenes**								
**101**	C_6H_5Cl	3.472	1.062	171.1	103.9	-0.02	4.31	24.70
**102**	C_6H_5Br	3.141	0.932	176.1	118.8	2.16	0.71	25.31
**103**	C_6F_5Cl	2.941	0.899	179.7	165.5	-0.96	-3.66	14.86
**104**	C_6F_5Br	2.866	0.850	179.7	147.0	3.90	-10.28	14.78
**105**	C_6F_5I	2.390	0.683	179.0	165.6	1.02	-58.38	-24.02
**Phosgene and its derivatives**								
**106**	$COCl_2$	2.916	0.892	177.7	150.5	2.85	-5.54	17.65
**107**	$COBr_2$	2.846	0.844	178.9	144.0	3.47	-10.85	15.60
**108**	COClF	2.908	0.889	179.3	179.7	2.98	-7.03	13.65
**109**	COBrF	2.867	0.851	178.8	164.4	2.11	-12.44	8.91
**110**	COBrCl	2.862	0.849	178.7	149.9	4.17	-10.64	13.32
**111**	COIF	2.356	0.673	179.6	172.2	1.04	-75.57	-44.27
**Thionyl- and sulfurylhalides**								

**112**	SO_2Cl_2	2.711	0.829	177.4	135.1	6.05	-12.67	14.96
**113**	SO_2**ClF**	2.732	0.836	178.9	144.5	6.39	-13.89	12.59
**114**	SO_2**Br**F	2.626	0.779	178.5	130.6	13.40	-24.85	3.59
**115**	$SOCl_2$	2.933	0.897	176.3	128.5	2.61	-2.81	20.30
**116**	$SOBr_2$	2.837	0.842	176.5	127.2	7.43	-9.13	18.89
**Sulfur halides and sulfur hypohalites**								
**117**	SF_6	3.089	1.033	176.3	177.1	-1.30	3.95	13.83
**118**	SF_5**Cl**	2.644	0.808	179.4	124.4	9.77	-13.02	12.83
**119**	SF_5**Br**	2.513	0.746	179.4	121.3	19.74	-28.64	3.53
**120**	SF_5**OF**	2.758	0.922	179.7	125.0	1.53	2.25	19.04
**121**	SF_5**OCl**	2.227	0.681	176.8	114.3	28.41	-38.09	2.33
**122**	S_2Cl_2	2.932	0.897	177.9	120.7	4.13	-0.98	23.14
**123**	S_2Br_2	2.800	0.831	178.6	123.2	8.25	-8.65	20.28
**124**	SCl_2	2.876	0.879	177.9	126.6	2.69	-3.69	20.75
**125**	SBr_2	2.758	0.818	177.2	125.0	7.03	-12.38	18.53
**Halogenated nitrogen-containing inorganic compounds**								
**126**	NF_3	3.041	1.017	173.7	147.5	0.43	3.88	20.33
**127**	NCl_3	2.639	0.807	176.0	123.0	9.23	-11.37	17.09
**128**	NI_3	2.330	0.666	158.0	97.8	36.98	-209.85	-154.85
**129**	NF_2**Cl**	2.651	0.811	178.1	125.4	8.99	-13.63	15.76
**130**	NF_2**Br**	2.591	0.769	178.2	123.6	14.51	-24.21	9.00
**131**	NO_2OF	2.843	0.951	178.8	115.2	1.75	3.15	27.77
**132**	NO_2OCl	2.360	0.722	177.7	116.1	19.41	-27.01	10.63
**133**	NO_2OBr	2.324	0.690	177.1	118.0	27.24	-48.17	-9.23
**134**	NO_2Cl	2.639	0.807	179.0	121.1	10.20	-10.49	19.75
**135**	NO_2Br	2.574	0.764	179.3	121.8	16.01	-22.09	11.97
**136**	NOBr	3.285	0.975	178.5	113.1	1.21	4.32	26.50
**Assorted organic compounds**								
**137**	$C_6H_5C_2Cl$	2.937	0.898	179.6	157.8	0.10	-3.77	18.58
**138**	$C_6H_5C_2Br$	2.872	0.852	179.1	142.7	4.34	-10.22	13.24
**139**	$C_6H_5C_2I$	2.405	0.687	179.0	164.8	-1.78	-58.41	-20.00
**140**	N-Chlorosuccinimide	2.686	0.821	171.3	115.3	6.89	-11.50	22.73
**141**	N-Bromosuccinimide	2.574	0.764	176.2	119.3	14.65	-23.63	7.76
**142**	N-Iodosuccinimide	2.328	0.665	175.0	104.5	27.39	-108.57	-61.35
**143**	Tetrachloro-1,4-benzoquinone	2.961	0.906	167.6	117.3	3.58	-6.16	25.07
**144**	Tetrabromo-1,4-benzoquinone	2.866	0.850	175.4	125.3	5.42	-11.62	17.24
**145**	Tetraiodo-1,4-benzoquinone	2.642	0.755	162.5	98.8	20.98	-138.62	-85.11

Table S2. Results of the electron density QTAIM analysis of the X···O (X = F, Cl, Br and I) halogen bond for 145 intermolecular 1:1 complexes formed by Me$_3$PO with halogen-donating molecules: distances from oxygen atom to minima positions of electron density $d(ED_{\min})$ and molecular electrostatic potential $d(ESP_{\min})$ along the bond path and the distance between them Δd, molecular electrostatic potential $ESP(r_{BCP})$, electron density $\rho(r_{BCP})$, Laplacian of electron density $\nabla^2\rho(r_{BCP})$, local electron potential $V(r_{BCP})$ and kinetic $G(r_{BCP})$ energy densities and total electron energy density $K(r_{BCP})$ at critical point of type (3; –1). The complexes with additional Y···H (Y = N, O, F, Cl, Br or I; H are protons of methyl groups) contacts shorter than the sum of Bondi's van der Waals radii of corresponding atoms are marked in red color. In ambiguous cases the halogen atoms which participate in halogen bond formation are highlighted in bold.

№	Halogen donor	$d(ED_{\min})$, Å	$d(ESP_{\min})$, Å	Δd, Å	$ESP(r_{BCP})$, kJ/mol	$\rho(r_{BCP})$, e/Å^3	$\nabla^2\rho(r_{BCP})$, e/Å^5	$V(r_{BCP})$, kJ/(mol·Å^3)	$G(r_{BCP})$, kJ/(mol·Å^3)	$K(r_{BCP})$, kJ/(mol·Å^3)
					**Halogens**					
**1**	F_2	1.1922	1.0652	0.1270	31.65	0.210	3.357	-496.91	556.99	60.08
**2**	Cl_2	1.2293	1.0203	0.2090	202.55	0.200	2.493	-417.65	437.92	20.27
**3**	Br_2	1.2139	0.9935	0.2204	331.49	0.226	2.499	-471.45	465.39	-6.06
**4**	I_2	1.0380	0.8546	0.1834	1042.55	0.484	4.511	-1584.60	1206.91	-377.69
**5**	F^+	0.6919	0.7255	-0.0336	2603.91	1.868	6.991	-7070.22	4177.59	-2892.63
**6**	Cl^+	0.8819	0.7504	0.1315	2687.61	1.310	-1.676	-4489.13	2090.57	-2398.57
**7**	Br^+	0.9464	0.7747	0.1717	2584.57	1.016	1.942	-3538.27	1947.58	-1590.69
**8**	I^+	0.9811	0.7700	0.2111	2883.26	0.934	6.760	-3633.30	2437.88	-1195.41
					**Interhalides**					
**9**	**ClF**	1.1314	0.9459	0.1855	484.53	0.328	3.567	-768.06	711.81	-56.26
**10**	**ClF$_3$**	1.0967	0.9332	0.1635	540.38	0.374	3.834	-857.95	781.28	-76.67
**11**	**ClF$_5$**	1.1798	1.0033	0.1765	261.13	0.242	2.809	-519.15	517.70	-1.45
**12**	**BrF**	1.1401	0.9282	0.2119	645.51	0.335	3.346	-801.15	708.04	-93.11
**13**	**BrF$_3$**	1.1025	0.9118	0.1908	727.39	0.390	3.522	-918.58	783.01	-135.57
**14**	**BrF$_5$**	1.1719	0.9763	0.1955	394.33	0.259	2.792	-563.84	538.50	-25.34
**15**	**IF**	1.0729	0.8671	0.2059	1101.11	0.516	3.021	-1572.25	1063.74	-508.51
**16**	**IF$_3$**	1.0319	0.8350	0.1969	1375.57	0.628	2.993	-1993.46	1271.81	-721.65
**17**	**IF$_5$**	1.0634	0.8829	0.1805	860.91	0.442	4.041	-1257.84	1000.31	-257.52
**18**	**BrCl**	1.1912	0.9740	0.2172	414.65	0.254	2.745	-551.56	528.08	-23.48
**19**	**ICl**	1.0832	0.8866	0.1966	921.33	0.468	3.174	-1372.75	978.10	-394.65
**20**	**IBr**	1.0856	0.8914	0.1942	886.40	0.460	3.131	-1338.27	956.88	-381.39
**21**	**ICl$_3$**	1.0377	0.8483	0.1895	1244.32	0.591	3.069	-1821.25	1192.65	-628.60
**22**	**ClF$_5$**	1.3079	1.1248	0.1831	-58.27	0.123	2.076	-260.68	321.11	60.43
					**Oxohalides**					
**23**	OF_2	1.5166	1.1917	0.3249	-140.35	0.049	0.884	-86.67	124.55	37.88
**24**	Cl_2O	1.2028	0.9970	0.2059	278.33	0.228	2.780	-497.00	503.94	6.94
**25**	Br_2O	1.1846	0.9647	0.2199	455.27	0.263	2.850	-586.37	555.13	-31.24
**26**	ClO_2	1.3566	1.0777	0.2789	73.15	0.106	1.492	-201.14	237.64	36.50
**27**	BrO_2	1.2875	1.0256	0.2619	231.72	0.154	1.923	-305.24	329.36	24.12
**28**	**ClO$_2$F**	1.2597	1.0242	0.2355	217.68	0.168	2.117	-338.65	363.88	25.24
**29**	**BrO$_2$F**	1.1769	0.9560	0.2209	506.81	0.272	2.742	-573.72	538.85	-34.87
**30**	**IO$_2$F**	1.0353	0.8186	0.2167	1548.64	0.647	2.420	-2014.64	1229.71	-784.93
**31**	ClO_3OF	1.4478	1.1690	0.2789	-128.57	0.064	1.168	-121.14	167.94	46.79
**32**	BrO_3OF	1.4134	1.1565	0.2569	-115.47	0.074	1.353	-145.84	197.26	51.42
**33**	IO_3OF	1.3356	1.1364	0.1992	-53.24	0.114	1.961	-242.18	301.32	59.14
**34**	ClO$_3$O**Cl**	1.1086	0.9340	0.1746	559.19	0.367	3.752	-862.16	775.87	-86.29
**35**	ClO$_3$O**Br**	1.0959	0.8996	0.1963	849.97	0.423	3.625	-1052.05	859.21	-192.85
**36**	ClO$_3$O**I**	1.0510	0.8425	0.2085	1399.74	0.605	2.774	-1938.50	1224.16	-714.34
					**Pseudohalides**					
**37**	FCN	1.4963	1.1999	0.2963	-153.20	0.041	0.829	-77.36	114.90	37.54
**38**	ClCN	1.3372	1.0777	0.2596	75.48	0.098	1.565	-196.88	242.27	45.39
**39**	BrCN	1.2952	1.0367	0.2585	201.75	0.129	1.855	-263.44	302.18	38.74
**40**	ICN	1.0658	0.8861	0.1797	793.00	0.390	4.820	-1229.55	1057.76	-171.79
**41**	ClSCN	1.3084	1.0676	0.2408	78.46	0.134	1.808	-257.79	295.01	37.22
**42**	BrSCN	1.2375	1.0078	0.2297	285.20	0.199	2.286	-404.35	412.27	7.93
**43**	ISCN	1.0803	0.8893	0.1910	887.40	0.461	3.344	-1349.97	982.35	-367.62
**44**	ClNCO	1.2721	1.0359	0.2363	162.82	0.161	2.174	-332.88	366.20	33.32
**45**	BrNCO	1.2116	0.9803	0.2313	392.03	0.228	2.630	-496.33	489.86	-6.47
**46**	INCO	1.0721	0.8718	0.2003	1029.31	0.493	3.410	-1519.01	1072.93	-446.08
**47**	ClN_3	1.2936	1.0533	0.2403	112.07	0.146	1.962	-290.80	325.68	34.88

**48**	BrN_3	1.2303	0.9975	0.2328	319.03	0.208	2.402	-434.44	437.95	3.51
**49**	IN_3	1.0906	0.8933	0.1974	864.18	0.443	3.341	-1286.73	950.39	-336.34
**Halogenated methanes and their derivatives**										
**50**	CH_3Br	1.5547	1.1698	0.3850	-80.25	0.050	0.658	-70.00	95.49	25.49
**51**	CH_3I	1.6108	1.2491	0.3617	58.87	0.060	0.727	-86.45	110.07	23.62
**52**	CH_2Br_2	1.4293	1.1142	0.3151	-6.59	0.081	1.085	-131.85	165.64	33.79
**53**	CH_2ClBr	1.5375	1.1708	0.3667	-89.30	0.051	0.726	-76.92	105.16	28.23
**54**	CH_2BrF	1.4693	1.1343	0.3350	-40.89	0.069	0.915	-105.84	137.00	31.16
**55**	CH_2BrCl	1.4349	1.1171	0.3178	-13.39	0.079	1.057	-127.49	160.92	33.43
**56**	CH_2BrI	1.4116	1.1081	0.3035	18.18	0.092	1.189	-154.13	186.33	32.20
**57**	CH_2IF	1.5711	1.2079	0.3633	69.05	0.067	0.787	-97.83	121.23	23.40
**58**	CH_2ICl	1.5489	1.1883	0.3606	82.82	0.071	0.832	-107.06	130.00	22.93
**59**	CH_2IBr	1.5468	1.1904	0.3564	75.49	0.072	0.839	-108.00	131.11	23.11
**60**	$CHBr_3$	1.3682	1.0816	0.2866	58.71	0.103	1.383	-182.51	218.37	35.87
**61**	$CHBrCl_2$	1.3745	1.0851	0.2895	50.34	0.100	1.345	-175.93	211.55	35.62
**62**	$CHBrI_2$	1.3521	1.0764	0.2757	98.57	0.123	1.520	-223.27	251.35	28.08
**63**	$CHIF_2$	1.5217	1.1690	0.3527	98.88	0.077	0.894	-119.94	142.16	22.21
**64**	$CHICl_2$	1.5029	1.1584	0.3445	101.35	0.083	0.942	-129.91	151.57	21.66
**65**	$CHIBr_2$	1.1967	0.9845	0.2122	379.36	0.257	2.783	-640.37	575.94	-64.43
**66**	$CHIClF$	1.5121	1.1642	0.3479	98.38	0.080	0.920	-124.61	146.85	22.24
**67**	$CHIClBr$	1.5005	1.1584	0.3421	100.44	0.083	0.950	-131.28	152.95	21.67
**68**	CCl_4	1.3912	1.1086	0.2826	-15.84	0.082	1.250	-148.93	189.37	40.44
**69**	CI_4	1.1401	0.9589	0.1812	488.98	0.335	3.431	-918.10	774.34	-143.76
**70**	CF_3Cl	1.4026	1.1195	0.2831	-31.86	0.073	1.151	-132.36	171.93	39.57
**71**	CCl_3F	1.3994	1.1129	0.2866	-19.41	0.078	1.201	-140.98	180.86	39.89
**72**	CF_3Br	1.3528	1.0748	0.2781	74.88	0.103	1.437	-189.26	226.68	37.42
**73**	CBr_3F	1.3343	1.0629	0.2715	105.14	0.118	1.577	-219.44	254.63	35.19
**74**	CF_3I	1.0695	0.8951	0.1744	702.12	0.377	4.724	-1168.39	1018.30	-150.09
**75**	CI_3F	1.1518	0.9538	0.1979	502.20	0.316	3.192	-835.62	711.20	-124.42
**76**	CCl_2F_2	1.3989	1.1163	0.2826	-27.67	0.075	1.179	-137.05	176.92	39.87
**77**	CBr_2F_2	1.3446	1.0692	0.2754	89.15	0.111	1.502	-203.96	240.04	36.08
**78**	CI_2F_2	1.1592	0.9576	0.2016	485.92	0.304	3.153	-799.21	689.41	-109.80
**79**	$CBrClF_2$	1.3460	1.0703	0.2757	85.71	0.109	1.489	-200.80	237.25	36.44
**80**	$CIClF_2$	1.0700	0.8938	0.1762	715.48	0.381	4.610	-1177.68	1012.50	-165.18
**81**	CF_3OF	1.4788	1.1843	0.2945	-133.38	0.059	1.050	-107.10	150.01	42.91
**82**	CF_3OCl	1.1436	0.9562	0.1873	445.89	0.308	3.407	-706.02	666.08	-39.94
**83**	CF_3OBr	1.1287	0.9224	0.2064	690.48	0.355	3.392	-849.35	736.43	-112.92
**84**	CF_3SO_2OF	1.3827	1.1502	0.2326	-102.12	0.089	1.563	-177.49	232.39	54.90
**85**	CF_3SO_2OCl	1.0472	0.8925	0.1548	800.81	0.503	4.244	-1244.82	1012.43	-232.40
**86**	CF_3SO_2OBr	1.0740	0.8824	0.1916	977.86	0.480	3.737	-1233.70	960.27	-273.43
**Halogenated ethylene, halogenated acetylene and their derivatives**										
**87**	C_2H_2IF	1.6304	1.2666	0.3638	72.15	0.057	0.692	-79.25	103.19	23.94
**88**	C_2H_2ICl	1.6243	1.2579	0.3665	71.54	0.058	0.696	-80.13	104.00	23.87
**89**	C_2H_2IBr	1.6153	1.2483	0.3670	70.81	0.059	0.707	-82.34	106.14	23.80
**90**	CI(H)C(F)Cl	1.5312	1.1690	0.3622	102.30	0.075	0.885	-116.47	139.53	23.07
**91**	CI(F)C(H)Cl	1.5328	1.1745	0.3583	93.31	0.074	0.865	-113.43	136.24	22.81
**92**	CI(Cl)C(H)F	1.5235	1.1679	0.3556	99.30	0.077	0.896	-119.81	142.25	22.43
**93**	C_2Cl_4	1.4177	1.1245	0.2932	-44.04	0.070	1.100	-124.54	163.29	38.75
**94**	C_2Br_4	1.3587	1.0758	0.2829	72.27	0.104	1.432	-188.97	226.04	37.08
**95**	C_2I_4	1.1343	0.9414	0.1929	560.35	0.345	3.537	-971.15	810.59	-160.56
**96**	C_2F_3Cl	1.4161	1.1245	0.2916	-41.07	0.069	1.101	-124.32	163.35	39.03
**97**	C_2F_3Br	1.3621	1.0793	0.2829	65.29	0.097	1.389	-178.45	216.85	38.41
**98**	C_2F_3I	1.0782	0.9030	0.1752	647.47	0.356	4.652	-1094.20	974.67	-119.53
**99**	$C_2(CN)_3Cl$	1.3288	1.0692	0.2596	91.68	0.113	1.669	-223.20	264.95	41.75
**100**	C_2Cl_2	1.3999	1.1147	0.2852	-21.75	0.073	1.187	-136.02	177.07	41.06
**Halogenated benzenes**										
**101**	C_6H_5Cl	1.7063	1.2221	0.4842	-108.39	0.028	0.379	-35.86	52.74	16.88
**102**	C_6H_5Br	1.4931	1.1441	0.3490	-56.41	0.062	0.831	-93.52	123.14	29.62
**103**	C_6F_5Cl	1.4137	1.1256	0.2881	-43.85	0.069	1.099	-123.95	162.93	38.98
**104**	C_6F_5Br	1.3573	1.0769	0.2805	70.80	0.099	1.415	-183.36	221.69	38.33
**105**	C_6F_5I	1.0869	0.9089	0.1781	620.75	0.346	4.419	-1042.77	927.47	-115.30
**Phosgene and its derivatives**										
**106**	$COCl_2$	1.4023	1.1174	0.2850	-27.22	0.076	1.167	-136.40	175.48	39.08
**107**	$COBr_2$	1.3449	1.0724	0.2725	83.65	0.107	1.469	-197.71	233.82	36.12
**108**	$COClF$	1.3939	1.1166	0.2773	-20.83	0.075	1.181	-137.35	177.18	39.82
**109**	$COBrF$	1.3499	1.0777	0.2723	74.31	0.098	1.411	-183.01	221.20	38.19
**110**	$COBrCl$	1.3507	1.0766	0.2741	73.30	0.102	1.423	-187.84	224.68	36.84

**111**	COIF	1.0713	0.8972	0.1741	694.09	0.373	4.694	-1153.58	1008.19	-145.39
**Thionyl- and sulfurylhalides**										
**112**	SO_2Cl_2	1.3095	1.0679	0.2416	78.56	0.127	1.760	-244.98	284.28	39.30
**113**	SO_2**ClF**	1.3179	1.0705	0.2474	74.72	0.117	1.692	-228.08	269.53	41.46
**114**	SO_2**Br**F	1.2515	1.0147	0.2368	264.24	0.180	2.163	-361.62	379.63	18.02
**115**	$SOCl_2$	1.4111	1.1266	0.2844	-48.46	0.081	1.139	-137.22	173.25	36.03
**116**	$SOBr_2$	1.3399	1.0766	0.2633	70.93	0.119	1.480	-210.60	241.35	30.75
**Sulfur halides and sulfur hypohalites**										
**117**	SF_6	1.6153	1.2383	0.3770	-199.13	0.024	0.462	-39.29	62.15	22.85
**118**	SF_5**Cl**	1.2835	1.0533	0.2302	114.47	0.150	1.987	-296.16	330.74	34.57
**119**	SF_5**Br**	1.2047	0.9872	0.2175	368.31	0.234	2.561	-492.04	481.41	-10.63
**120**	SF_5**OF**	1.4648	1.1790	0.2858	-146.09	0.057	1.059	-105.99	150.31	44.32
**121**	SF_5**OCl**	1.0996	0.9271	0.1725	591.53	0.384	3.847	-912.82	809.94	-102.88
**122**	S_2Cl_2	1.4182	1.1290	0.2892	-49.20	0.082	1.137	-137.85	173.43	35.59
**123**	S_2Br_2	1.3285	1.0676	0.2609	92.50	0.128	1.581	-231.27	260.95	29.69
**124**	SCl_2	1.3917	1.1121	0.2797	-22.26	0.089	1.273	-157.24	195.63	38.39
**125**	SBr_2	1.3118	1.0541	0.2577	128.61	0.137	1.716	-256.31	285.87	29.57
**Halogenated nitrogen-containing inorganic compounds**										
**126**	NF_3	1.6042	1.2216	0.3826	-185.73	0.028	0.525	-45.04	70.74	25.69
**127**	NCl_3	1.2878	1.0491	0.2387	121.25	0.148	1.992	-297.03	331.59	34.57
**128**	NI_3	1.0835	0.8967	0.1868	845.52	0.459	3.375	-1348.87	984.59	-364.27
**129**	NF_2**Cl**	1.2917	1.0510	0.2408	119.32	0.144	1.947	-288.01	322.96	34.95
**130**	NF_2**Br**	1.2425	1.0065	0.2360	284.80	0.194	2.263	-397.60	406.78	9.18
**131**	NO_2OF	1.5177	1.1928	0.3249	-144.32	0.049	0.877	-85.77	123.50	37.72
**132**	NO_2OCl	1.1613	0.9684	0.1929	396.79	0.282	3.215	-636.61	613.74	-22.87
**133**	NO_2OBr	1.1330	0.9266	0.2064	668.17	0.347	3.341	-822.83	718.46	-104.37
**134**	NO_2Cl	1.2822	1.0536	0.2286	109.37	0.154	1.978	-301.79	332.64	30.85
**135**	NO_2Br	1.2314	1.0046	0.2268	292.81	0.206	2.313	-420.34	422.76	2.42
**136**	NOBr	1.5431	1.1811	0.3620	-108.86	0.053	0.632	-70.11	93.17	23.05
**Assorted organic compounds**										
**137**	$C_6H_5C_2Cl$	1.4158	1.1224	0.2934	-39.71	0.069	1.115	-125.41	165.16	39.75
**138**	$C_6H_5C_2Br$	1.3642	1.0771	0.2871	69.77	0.098	1.408	-180.61	219.69	39.08
**139**	$C_6H_5C_2I$	1.0930	0.9134	0.1797	586.46	0.334	4.372	-1002.87	903.22	-99.65
**140**	N-Chlorosuccinimide	1.3163	1.0613	0.2551	92.08	0.135	1.837	-265.10	301.41	36.31
**141**	N-Bromosuccinimide	1.2420	0.9996	0.2424	304.57	0.199	2.354	-416.55	424.61	8.06
**142**	N-Iodosuccinimide	1.0893	0.8914	0.1979	846.28	0.438	3.579	-1305.86	981.80	-324.05
**143**	Tetrachloro-1,4-benzoquinone	1.4378	1.1285	0.3093	-33.40	0.076	1.090	-129.48	164.91	35.43
**144**	Tetrabromo-1,4-benzoquinone	1.3640	1.0774	0.2866	76.96	0.107	1.426	-191.73	226.88	35.16
**145**	Tetraiodo-1,4-benzoquinone	1.2036	0.9933	0.2104	342.75	0.246	2.791	-612.50	562.71	-49.80

Figure S2. ED and ESP profiles along the halogen bond path (blue and red curves, respectively, where minima positions are indicated by vertical dashed lines) for (a) weak, (b) medium and (c) strong R–Cl···$OPMe_3$ complexes (complexes **[53]**, **[9]** and **[85]**, respectively; see Figure S1). The values of Δ*d* and $G(r_{BCP})$. for the Cl···O bonds are given at the bottom of the figure.

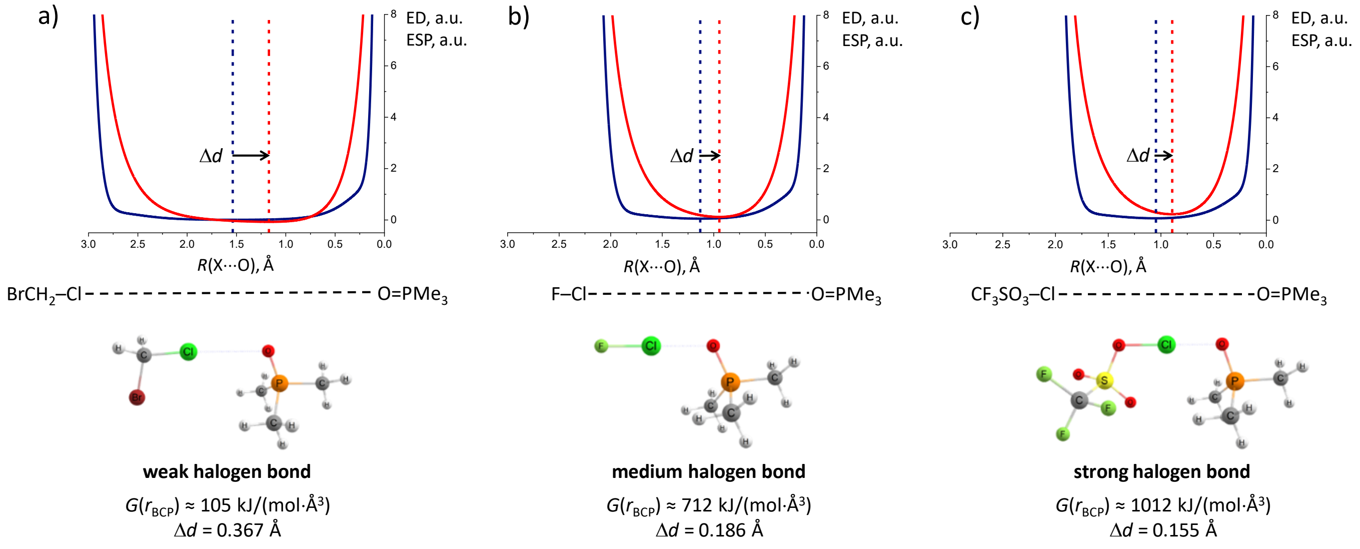

Figure S3. Optimized structures of (a) Me_3POF^+, (b) Me_3POCl^+, (c) Me_3POBr^+ and (d) Me_3POI^+ complexes. Blue dot marks the position of the X···O bond critical point. The values of $G(r_{BCP})$ and Δd for the X···O bonds are given.

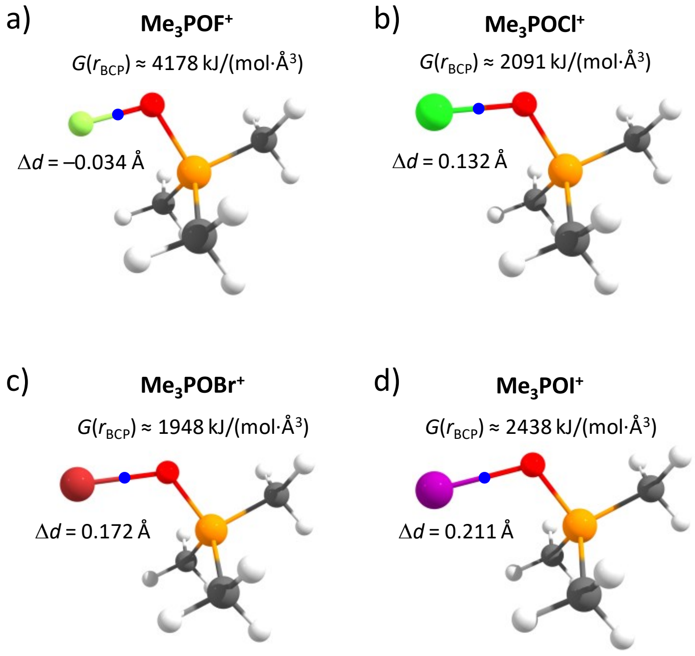

Figure S4. Complexation enthalpy ΔH as a function of $G(r_{BCP})$ at critical point of type (3; –1) of 145 intermolecular 1:1 complexes formed by Me_3PO with halogen-donating molecules. Open symbols correspond to complex with the presence of additional non-covalent interactions, filled symbols – without such interactions (halogen bond only).

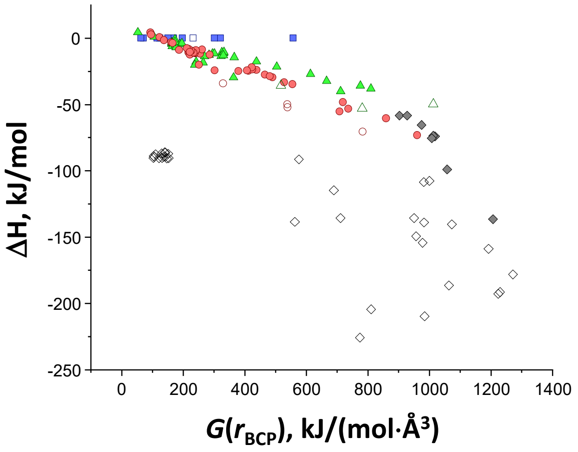

Figure S5. (a) Distances from oxygen atom to minima of electron density $d(ED_{min})$ and molecular electrostatic potential $d(ESP_{min})$ along the X···O (X = F, Cl, Br and I) bond path; (b) distances between ED and ESP minima Δd; (c) normalized X···O (X = F, Cl, Br and I) distances R_{norm} as a function of electron density $\rho(r_{BCP})$ at critical point of type (3; –1) of 145 intermolecular 1:1 complexes formed by Me_3PO with halogen-donating molecules.

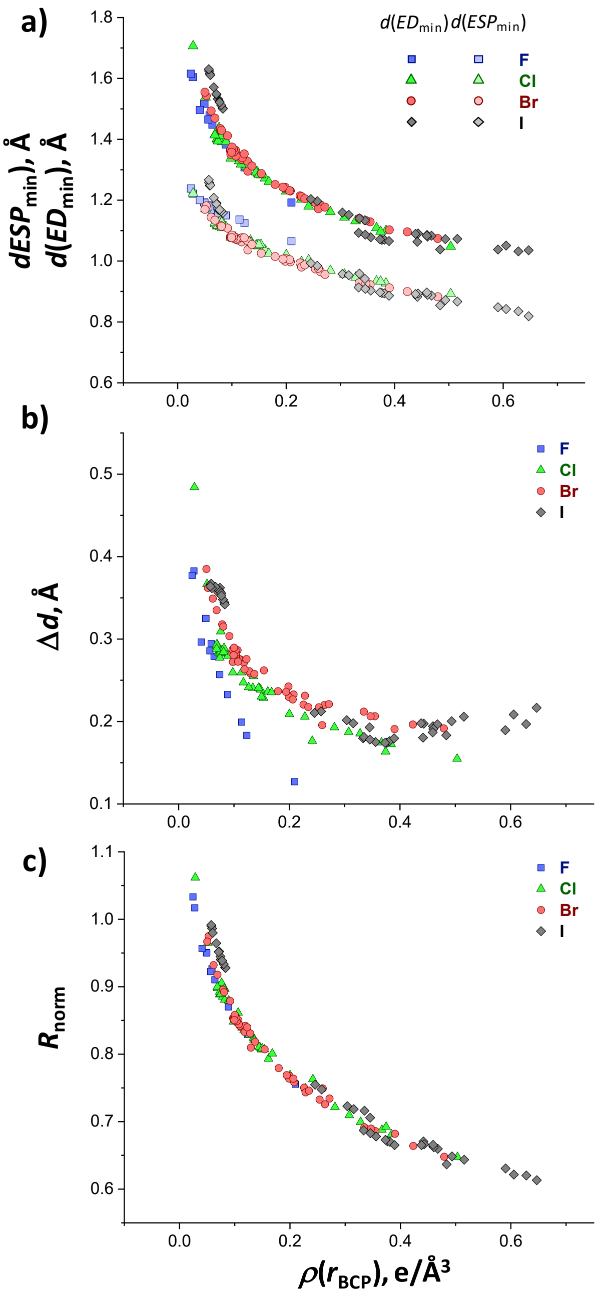

Figure S6. (a) Distances from oxygen atom to minima of electron density $d(ED_{min})$ and molecular electrostatic potential $d(ESP_{min})$ along the X···O (X = F, Cl, Br and I) bond path; (b) distances between ED and ESP minima Δd; (c) normalized X···O (X = F, Cl, Br and I) distances R_{norm} as a function of Laplacian of electron density $\nabla^2\rho(r_{BCP})$ at critical point of type (3; –1) of 145 intermolecular 1:1 complexes formed by Me_3PO with halogen-donating molecules.

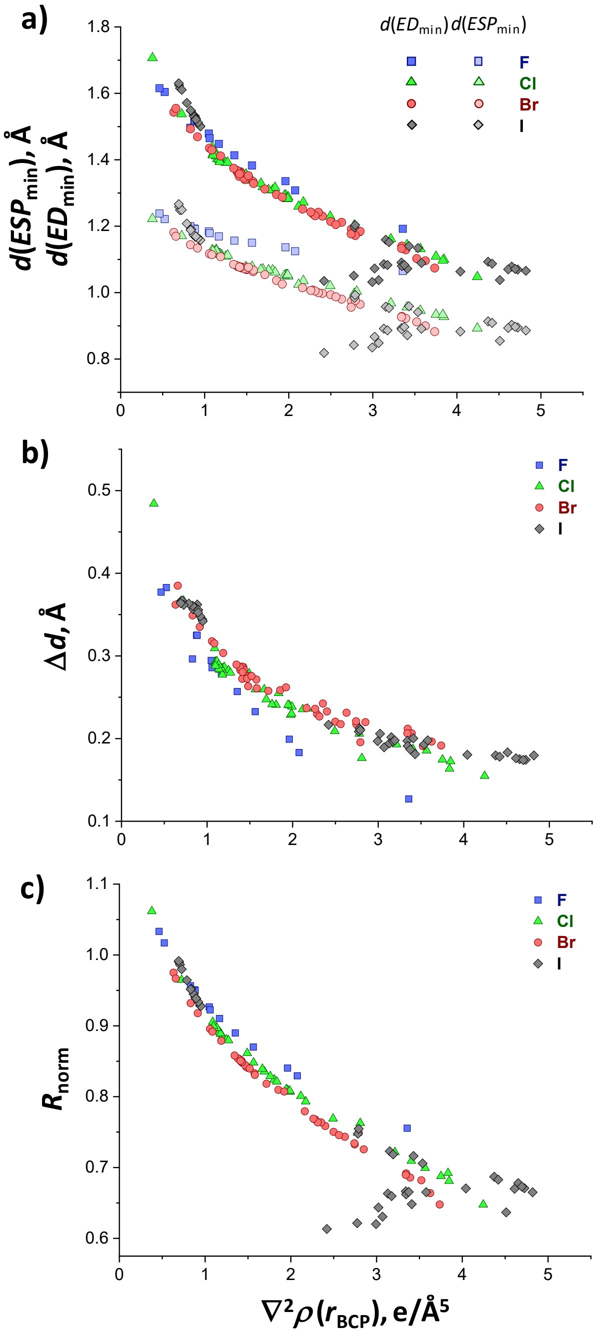

Figure S7. (a) Distances from oxygen atom to minima of electron density $d(ED_{min})$ and molecular electrostatic potential $d(ESP_{min})$ along the X···O (X = F, Cl, Br and I) bond path; (b) distances between ED and ESP minima Δd; (c) normalized X···O (X = F, Cl, Br and I) distances R_{norm} as a function of total electron energy density $K(r_{BCP})$ at critical point of type (3; –1) of 145 intermolecular 1:1 complexes formed by Me_3PO with halogen-donating molecules.

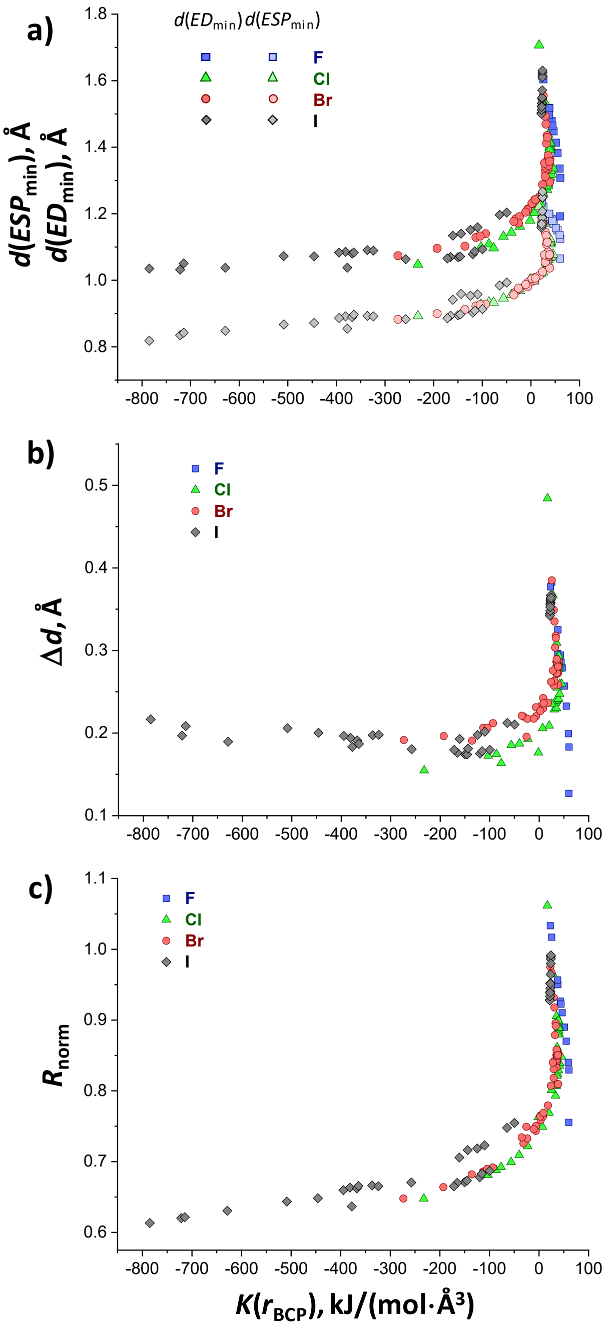

Figure S8. (a) Local electron potential energy density $V(r_{BCP})$, (b) electron density $\rho(r_{BCP})$, (c) Laplacian of electron density $\nabla^2\rho(r_{BCP})$, (d) molecular electrostatic potential $ESP(r_{BCP})$ at the X···O (X = F, Cl, Br and I) bond critical point of type (3; –1) as a function of local electron kinetic energy density $G(r_{BCP})$ of 145 intermolecular 1:1 complexes formed by Me_3PO with halogen-donating molecules.

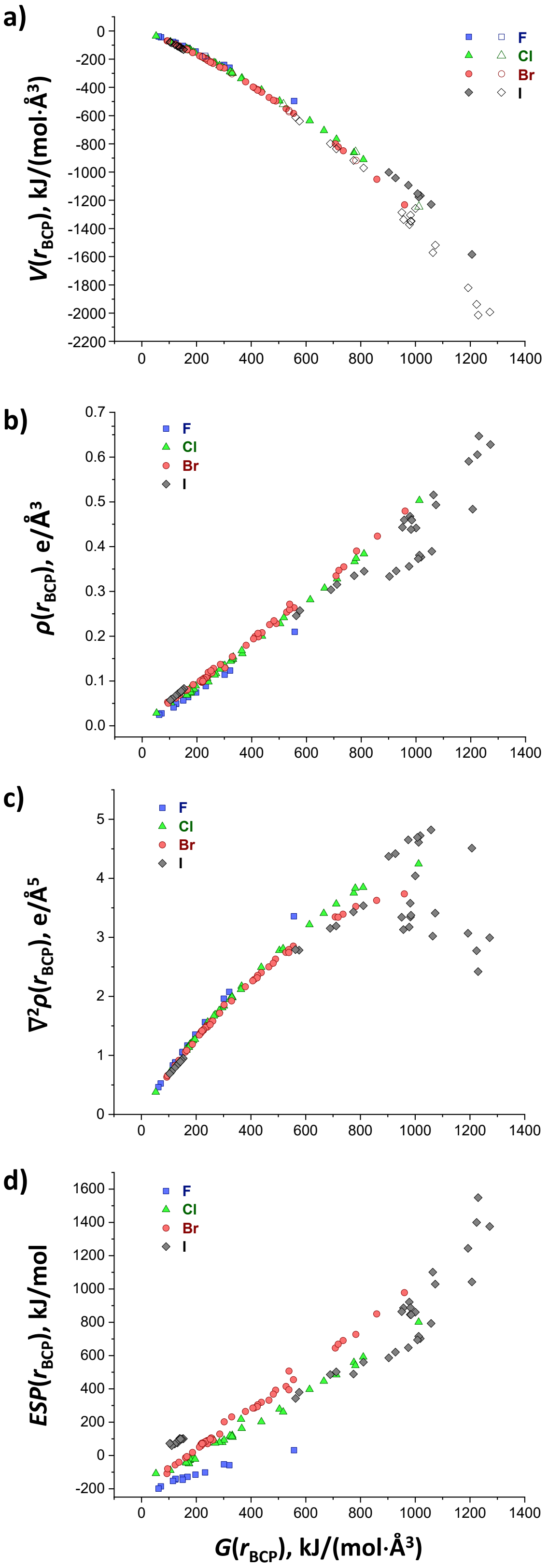

Figure S9. (a) Complexation enthalpy ΔH, (b) electron density $\rho(r_{BCP})$, (c) Laplacian of electron density $\nabla^2\rho(r_{BCP})$, (d) molecular electrostatic potential $ESP(r_{BCP})$ at the X···O (X = F, Cl, Br and I) bond critical point of type (3; –1) as a function of distance between ED and ESP minima Δd of 145 intermolecular 1:1 complexes formed by Me_3PO with halogen-donating molecules. Open symbols correspond to complex with the presence of additional non-covalent interactions, filled symbols – without such interactions (halogen bond only).

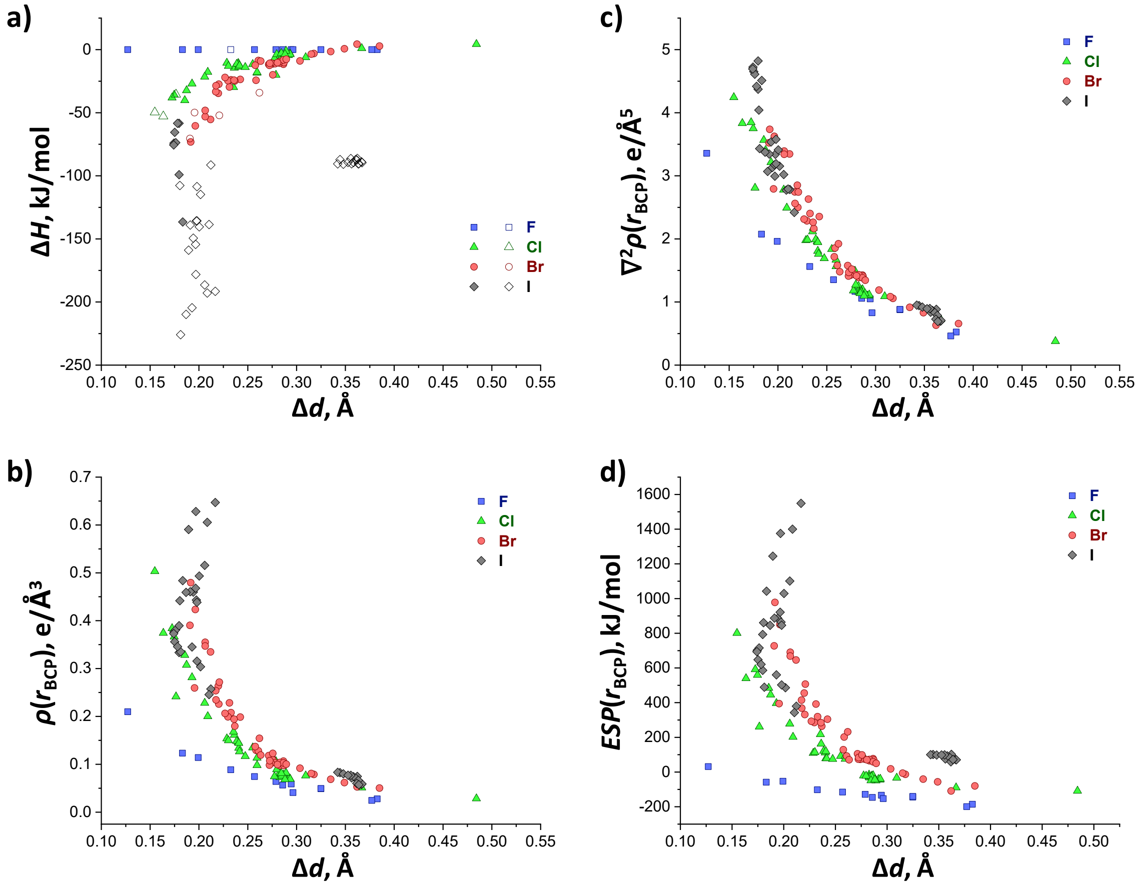

Figure S10. (a) Absolute, R(X···O), and (b) normalized, R_{norm}, values of X···O (X = F, Cl, Br and I) distances as a function of local electron kinetic energy density $G(r_{\text{BCP}})$ at critical point of type (3; –1) of 145 intermolecular 1:1 complexes formed by Me_3PO with halogen-donating molecules. The solid curve was obtained as described below the figure.

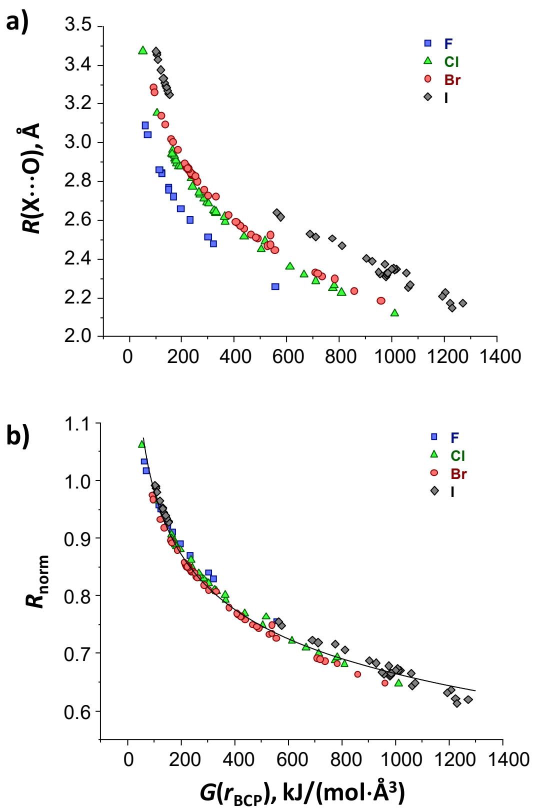

The R_{norm} values fall predictably with the increase of $G(r_{\text{BCP}})$, making the terms "halogen bond strength" and "halogen bond shortness" virtually interchangeable. By losing some precision and gaining some generality, one could propose the following halogen-independent fitting curve for the whole set of data points in Figure S10:

$$R_{\text{norm}} = -0.784 \cdot \ln(0.062 \cdot \ln G(r_{\text{BCP}}))$$

This functional dependence is clearly lacking a physical meaning, but it described the data points fairly and thus might appear to be useful.

Figure S11. (a) Complexation enthalpy ΔH and (b) local electron kinetic energy density $G(r_{BCP})$ at the X···O (X = F, Cl, Br and I) bond critical point of type (3; –1) as a function of the change of the ^{31}P NMR chemical shift upon complexation $\Delta\delta^{31}$P of 145 intermolecular 1:1 complexes formed by Me_3PO with halogen-donating molecules. Open symbols correspond to complex with the presence of additional non-covalent interactions, filled symbols – without such interactions (halogen bond only).

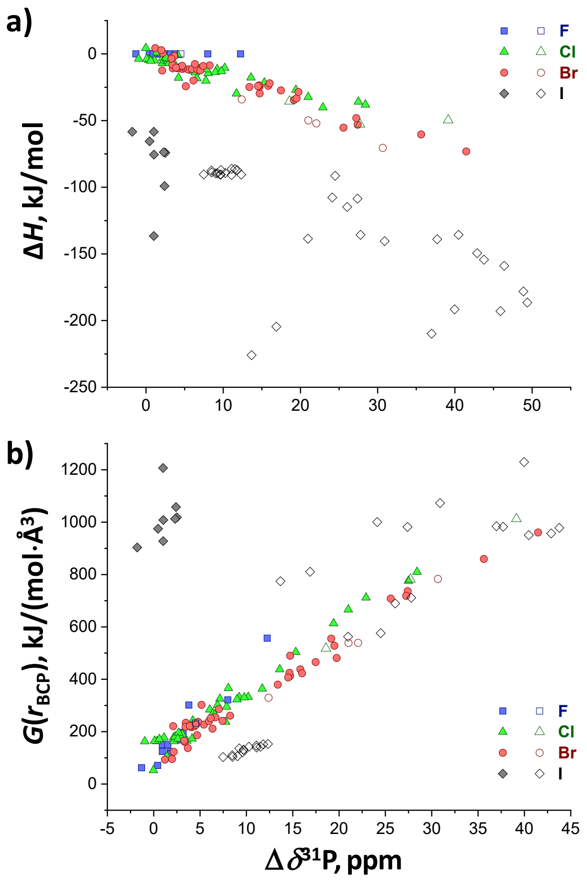

Supplement: Supplementary file 1 [file molecules-27-04848-s001.zip › molecules-1808161-supplementary.pdf]
